# Innovative Strategies for Combating Multidrug-Resistant Tuberculosis: Advances in Drug Delivery Systems and Treatment

**DOI:** 10.3390/microorganisms13040722

**Published:** 2025-03-24

**Authors:** Omobolanle A. Omoteso, Adewale O. Fadaka, Roderick B. Walker, Sandile M. Khamanga

**Affiliations:** 1 Division of Pharmaceutics, Faculty of Pharmacy, Rhodes University, Makhanda 6139, South Africa; r.b.walker@ru.ac.za (R.B.W.); s.khamanga@ru.ac.za (S.M.K.); 2 Department of Anesthesia, Division of Pain Management, Cincinnati Children’s Hospital Medical Center, Cincinnati, OH 45229, USA; adewale.fadaka@cchmc.org

**Keywords:** multidrug-resistant tuberculosis, drug delivery systems, nanotechnology, drug resistance mechanisms, host-directed therapies

## Abstract

Multidrug-resistant tuberculosis (MDR-TB) is a significant public health challenge globally, exacerbated by the limited efficacy of existing therapeutic approaches, prolonged treatment duration, and severe side effects. As drug resistance continues to emerge, innovative drug delivery systems and treatment strategies are critical to combating this crisis. This review highlights the molecular mechanisms underlying resistance to drugs in *Mycobacterium tuberculosis*, such as genetic mutation, efflux pump activity, and biofilm formation, contributing to the persistence and difficulty in eradicating MDR-TB. Current treatment options, including second-line drugs, offer limited effectiveness, prompting the need for innovation of advanced therapies and drug delivery systems. The progression in drug discovery has resulted in the approval of innovative therapeutics, including bedaquiline and delamanid, amongst other promising candidates under investigation. However, overcoming the limitations of traditional drug delivery remains a significant challenge. Nanotechnology has emerged as a promising solution, with nanoparticle-based drug delivery systems offering improved bioavailability and targeted and controlled release delivery, particularly for pulmonary targeting and intracellular delivery to macrophages. Furthermore, the development of inhalable formulations and the potential of nanomedicines to bypass drug resistance mechanisms presents a novel approach to enhancing drug efficacy. Moreover, adjunctive therapies, including immune modulation and host-directed therapies, are being explored to improve treatment outcomes. Immunotherapies, such as cytokine modulation and novel TB vaccines, offer complementary strategies to the use of antibiotics in combating MDR-TB. Personalized medicine approaches, leveraging genomic profiling of both the pathogen and the host, offer promise in optimizing treatment regimens and minimizing drug resistance. This review underscores the importance of multidisciplinary approaches, combining drug discovery, advanced delivery system development, and immune modulation to address the complexities of treating MDR-TB. Continued innovation, global collaboration, and improved diagnostics are essential to developing practical, accessible, and affordable treatments for MDR-TB.

## 1. Introduction

Tuberculosis (TB) continues to pose a formidable challenge to public health in the 21st century [1]. Despite being a preventable and treatable disease, it has resulted in approximately 1.6 million fatalities worldwide in 2021, positioning it as the second leading cause of death from a singular infectious agent following COVID-19 [2]. The rise of multidrug-resistant tuberculosis (MDR-TB) has compounded this burden, undermining decades of progress in the treatment and control of TB [3]. MDR-TB is characterized by resistance to at least isoniazid and rifampin, the foremost first-line anti-TB medicines, and accounts for approximately 450,000 new cases annually, with treatment success rates of approximately 60% [4]. The emergence and persistence of MDR-TB are driven by a combination of factors, including incomplete or incorrect treatment regimens, genetic adaptability of *Mycobacterium tuberculosis (Mtb),* and the lack of access to timely and accurate diagnostic tools [4]. These challenges necessitate innovative strategies to improve the efficacy of existing treatments and the delivery of novel therapeutic agents. Recent advances in molecular biology, drug development, and drug delivery systems offer a glimmer of hope in the fight against MDR-TB [5,6,7,8]. However, successfully translating these innovations into widespread clinical practice remains formidable.

This review provides an in-depth overview of the latest advances in managing MDR-TB, focusing on cutting-edge drug delivery systems (DDS) and innovative drug therapies. By highlighting both the successes and challenges of these approaches, we seek to identify opportunities for future research and implementation that can bridge the gap between scientific discovery and clinical impact.

MDR-TB is part of a broader spectrum of drug-resistant TB, which includes extensively drug-resistant TB (XDR-TB) and, more recently, totally drug-resistant TB (TDR-TB) [9]. XDR-TB is characterized by resistance to fluoroquinolones and a minimum of one second-line injectable drug and has been reported in over 120 countries [10]. The management of drug-resistant TB incurs significant costs, estimated to be nearly 20 times greater than those associated with drug-susceptible TB. However, it is also associated with more severe side effects and prolonged treatment duration [10]. One alarming statistic is the high mortality rate associated with untreated or poorly treated MDR-TB, which can reach up to 80% [11]. Furthermore, MDR-TB often affects individuals in low- and middle-income nations, where healthcare infrastructure is ill-equipped to manage complex drug regimens [12]. These realities underscore the urgent need for improved diagnostic, therapeutic, and preventive measures.

In recent years, several novel pharmaceuticals have received approval for treating MDR-TB, including bedaquiline, delamanid, and pretomanid. These drugs have shown promise in reducing treatment duration and improving clinical outcomes, mainly when used as part of an all-oral regimen [13]. For instance, a recent study demonstrated that a combination of bedaquiline, pretomanid, and linezolid (the BPaL regimen) achieved an 89% treatment success rate when treating individuals infected with XDR-TB [13]. In addition, repurposing existing drugs, such as clofazimine and meropenem, has expanded treatment options for combatting MDR-TB. These strategies provide alternative options and help mitigate the risk of further development of resistance. However, challenges such as drug toxicity, high costs, and limited availability persist, highlighting the need for additional innovation [14,15].

Conventional TB treatment relies on systemic drug administration, which often leads to suboptimal drug concentrations at the site(s) of infection, thereby contributing to treatment failure and resistance [16]. Innovative drug delivery systems, such as nanoparticles, liposomes, and inhalation formulations, have emerged as potential game-changers. These technologies facilitate precise drug delivery, prolonged release, and enhanced bioavailability, augmenting therapeutic efficacy and minimizing side effects. For example, inhaled formulations of anti-TB drugs have been shown to deliver drugs in high concentrations directly to the lungs, the primary site of infection. This approach reduces systemic toxicity and shortens treatment duration in preclinical models [17]. Furthermore, delivery systems rooted in nanotechnology, including polymeric nanoparticles and lipid-based carriers, have shown significant promise in surmounting biological barriers and facilitating the delivery of drugs to the intracellular reservoirs of *Mtb* [18,19]. While the advances in treatment and drug delivery systems are promising, their impact on the global MDR-TB epidemic will depend on equitable access, robust healthcare infrastructure, and effective policy implementation. Investment in research and development must complement strategies to ensure affordability and accessibility, particularly in high-burden settings.

Moreover, integrating novel diagnostics, such as rapid molecular tests and whole-genome sequencing, into TB programs can facilitate the timely identification of drug resistance and guide the development of personalized treatment regimens [20]. Such a comprehensive approach is essential to curbing the spread of MDR-TB and achieving the goals of the End TB Strategy by 2030 [2]. This review analyzes the distinctive features of MDR-TB and investigates the latest developments in treatment and drug delivery approaches. The discussion includes emerging drugs, innovative drug delivery mechanisms, and immune modulation as central focus areas.

## 2. Peculiarities of TB and Mechanisms of MDR-TB

### 2.1. Tuberculosis Pathophysiology and Drug Resistance Mechanisms

**Figure 1:**
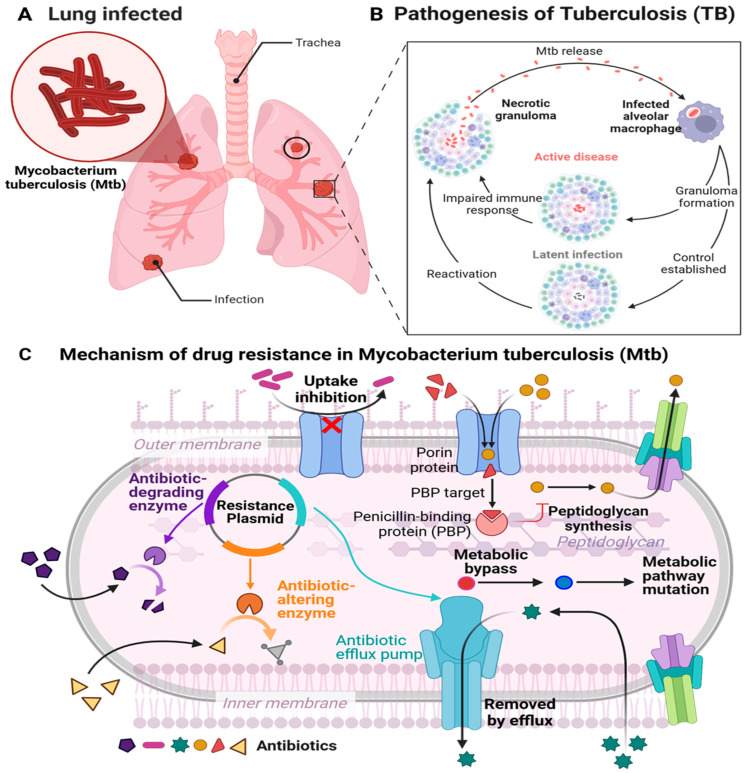
Overview of tuberculosis pathogenesis and mechanisms of resistance to drug by *Mycobacterium tuberculosis (Mtb)*. (**A**) Lung infection following contact with *Mtb*. The bacteria infect alveolar macrophages in the lungs, initiating the disease process. (**B**) Pathogenesis of tuberculosis. Granuloma formation occurs as a host defense mechanism, transitioning between active diseases, where *Mtb* is released and the immune response is impaired, and latent infection, where granulomas control bacterial growth. Reactivation can occur on immune suppression. (**C**) Mechanisms of drug resistance in *Mtb.* Resistance plasmids mediate antibiotic resistance by producing antibiotic-degrading enzymes, antibiotic-altering enzymes, and efflux pumps. Mutations in porin proteins reduce antibiotic entry while penicillin-binding protein (PBP) mutations disrupt antibiotic targeting of peptidoglycan synthesis. Metabolic pathway mutations and bypasses further enable bacterial survival under antibiotic pressure. Created with BioRender.com.

TB is a severe infectious disease that primarily impacts the pulmonary system. Moreover, it can affect other body parts, including the kidneys, spine, bones, lymph nodes, and brain, and is attributed to *Mtb* (Figure 1A), a highly specialized human pathogen [21,22,23].

#### 2.1.1. Pathophysiology of TB

*Mtb* transmission occurs via airborne particles expelled by individuals who cough, sneeze, or spit, thereby distributing the infectious organism. The bacteria can persist in the air for several hours, rapidly infecting others [24,25]. Following inhalation, *Mtb* is primarily engulfed by resident alveolar macrophages in the lungs [25]. Macrophages internalize pathogens via phagocytosis, which is facilitated by ligand–receptor interactions involving mannose receptors, scavenger receptors, complement receptors (CR1, CR3, CR4), Fc receptors, and surfactant protein receptors [26,27]. *Mtb* skillfully circumvents the bactericidal strategies of macrophages through mechanisms that include the prevention of bacterial vacuole acidification, inhibition of phagolysosome formation, and the obstruction of apoptosis and autophagy in infected macrophages [26,28]. After internalization, *Mtb* inhibits phagosome maturation, allowing it to merge with lysosomes and persist within macrophages. Alternatively, it replicates intracellularly by preventing phagosome–lysosome fusion and transforming macrophages into a protective environment for the pathogen [27,29]. After infection of alveolar macrophages within the lower respiratory tract, *Mtb* infiltrates the interstitium of the lung(s), thereby advancing the course of the infection process. The incursion of pathogens into the parenchyma triggers an immune response, resulting in the mobilization of T and B cells to the locus of infection. This recruitment precipitates a complex multicellular reaction by the host called granulomatous inflammation. Granulomas serve the purpose of efficiently confining the *Mtb* pathogen at the site(s) of infection, thus inhibiting spreading in the host. Nonetheless, the progression of dysfunctional granulomas may lead to the persistence of pathogens, considerable tissue damage, and insufficient responses to treatment. *Mtb* utilizes granulomas (Figure 1B) as focal points of infection, where phagocytic cells congregate, providing ample nutrients for replication [26,27]. Infected individuals who do not present with disease symptoms cannot transmit *Mtb*; however, the bacteria persist within the body in an inactive or latent form, which, if left untreated, can advance to active TB (Figure 1B) [25].

#### 2.1.2. Conventional Anti-Tubercular Pharmaceuticals

TB is typically managed using antibiotics, including isoniazid (INH), rifampin (RIF), pyrazinamide (PZA), ethambutol hydrochloride (EMB), and streptomycin (STREP) (Table 1), and can be deadly if left untreated. The existing treatment protocol for tuberculosis involves a daily dosing regimen for between four and six months. This treatment involves an intensive two-month phase, during which patients are administered four first-line antibiotics, viz., EMB, INH, RIF, and PZA. This is followed by a four-month continuous phase of treatment, where the regimen includes RIF and INH [24]. When bacteria resist standard medications, it may become necessary to use second-line treatment, such as injectable formulations of amikacin, capreomycin, and kanamycin, thereby leading to more intricate and extended therapeutic processes that frequently exhibit reduced efficacy, higher costs, and more pronounced adverse effects [24,30]. Fluoroquinolones, including moxifloxacin (MOX), gatifloxacin, levofloxacin, and ofloxacin in combination with other oral agents such as para-aminosalicylic acid, prothionamide, terizidone, cycloserine, and ethionamide, are used for the treatment of TB [26,31].

### 2.2. Mechanism of Action of Anti-Tubercular Agents

**Figure 2:**
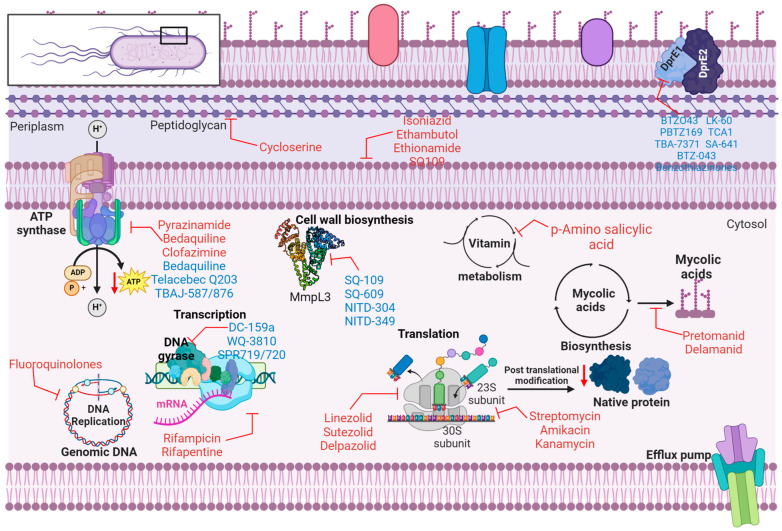
Mechanisms of action of anti-tuberculosis drugs targeting *Mycobacterium tuberculosis*. The sites of action for anti-tuberculosis medicines targeting *Mycobacterium tuberculosis*. Drugs such as pyrazinamide, bedaquiline, and clofazimine inhibit ATP synthesis, while isoniazid, ethambutol, ethionamide, and cycloserine target the synthesis of the mycobacterial cell wall. Rifampicin and rifapentine inhibit transcription by blocking RNA polymerase. Fluoroquinolones act on DNA replication, while linezolid, sutezolid, and delpazolid interfere with translation. Streptomycin, amikacin, and kanamycin disrupt post-translational modifications. Additional drugs such as pretomanid and delamanid inhibit mycolic acid biosynthesis, and p-aminosalicylic acid impairs folate metabolism. Red: Approved Drugs for Tuberculosis Treatment, Blue: Investigational Drugs for Tuberculosis Treatment. Created with BioRender.com.

Isoniazid interacts with two specific proteins in *Mtb*. The primary target is catalase-peroxidase, an enzyme that protects the organism against reactive oxygen species activity. The second target is enoyl-acyl-carrier-protein reductase, which is involved in mycolic acid production. *Mtb* inhibits the biosynthesis of mycolic acid, impacting the cell wall (Table 1 and Figure 2), and affects the metabolism of DNA, lipids, carbohydrates, and NAD. Rifampin interacts with and obstructs DNA-dependent RNA polymerase, thereby impeding RNA synthesis. Ethambutol exhibits bacteriostatic properties by inhibiting arabinosyl transferase A, B, and C, disrupting cell wall synthesis. Pyrazinamide inhibits *Mtb* fatty acid synthetase, leading to plasma membrane disruption and intracellular acidification, impairing energy metabolism. Rifapentine is an antibiotic that similarly inhibits DNA-dependent RNA polymerase, similar to rifampicin. Streptomycin targets the S12 and 16S rRNA components of the 30S ribosomal subunit, inhibiting protein synthesis. Similarly, amikacin and kanamycin also target the 30S ribosomal subunit and inhibit protein synthesis. Para-amino salicylic acid inhibits folic acid and iron metabolism by targeting thymidylate synthase (*ThyA*) and dihydropteroate synthase. Fluoroquinolones target DNA gyrase and DNA topoisomerase, leading to the inhibition of DNA supercoiling. Cycloserine inhibits D-alanine racemase and ligase, thereby disrupting peptidoglycan biosynthesis. Ethionamide inhibits Enoyl-[acyl-carrier protein] reductase (InhA), disrupting mycolic acid biosynthesis. Capreomycin targets Interbridge B2a and inhibits protein synthesis [27,31,32]. Some investigational drugs for the treatment of TB are listed in Table 2.

### 2.3. Mechanism of Drug Resistance (MDR-TB)

The increase in strains resistant to widely used anti-TB medications has created a quandary for researchers [33]. Understanding the principal factors contributing to drug-resistant tuberculosis in clinical instances, such as genetic changes in drug targets or activation enzymes, the synthesis of drug-inactivating enzymes, compensatory evolution, and the activation of efflux pumps on the bacterial surface [30,51]. Moreover, spontaneous mutations in the *Mtb* genome might modify enzymes/proteins that change the affinity of antibiotics for their target, rendering the bacteria resistant to the medicine contingent on the drug’s mechanism of action (Figure 1C).

#### 2.3.1. Genetic Mechanisms of Resistance

The primary mechanism behind the development of MDR-TB is the occurrence of specific genetic mutations in *Mtb* that confer first-line antibiotic resistance. Mycobacterium genomes that exhibit mutations and various structural alterations can resist the effects of commonly used drugs that inhibit growth.

- Isoniazid Resistance

Isoniazid, classified as a prodrug, undergoes activation through the catalase-peroxidase enzyme, expressed by the *katG* gene. Alterations in the *katG* gene, responsible for encoding the activating enzyme catalase-peroxidase, are essential for understanding the core mechanism(s) of resistance. These mutations represent a significant area of study and understanding in microbiology and pharmacology. Furthermore, alterations in the *inhA* gene, which is responsible for encoding the target enzyme for isoniazid, affect the target enzyme, enoyl-ACP reductase. Alterations in the *inhA* gene represent the second most common mechanism by which resistance occurs. The expression of untargeted proteins, a significant factor in resistance to treatment, underscores the urgency of addressing this issue. The impact of resistance to therapy is a key area of concern and research. These alterations undermine the efficacy of drugs in disrupting mycolic acid production, which is essential for mycobacterial cell wall integrity [31,33,52].

- Rifampicin Resistance

Rifampicin exhibits strong bactericidal properties and effectively sterilizes *Mtb*. Rifampicin is recognized for its ability to disrupt RNA synthesis by binding to the ß subunit of RNA polymerase. Changes in the *rpoB* gene, which encodes the beta-subunit of RNA polymerase, lead to modification of the β-subunit of RNA polymerase. The mutations hinder rifampicin binding to the enzyme, obstructing its inhibitory impact on bacterial transcription. Approximately 95% of rifampicin resistance is linked to mutations in an 81-base pair segment of *rpoB* between codons 507 and 533 [31,52,53]. The mutations that lead to resistance against these drugs frequently arise spontaneously, taking place during the replication of bacteria when exposed to suboptimal or incomplete treatment regimens. Mutations in the target genes of antibiotics are regarded as the primary resistance mechanism in this bacterium [4].

#### 2.3.2. Non-Genetic Mechanisms

- Efflux Pump Mechanisms

Drug resistance cannot solely be attributed to gene mutations in a subset of clinical *Mtb* strains. Previous studies indicate that 30% of isoniazid-resistant and 5% of rifampicin-resistant *Mtb* clinical strains exhibit this phenomenon, implying the existence of alternative mechanisms of drug resistance. Efflux has been identified as a potential mechanism underlying drug resistance in clinical strains that lack the previously described gene mutations [54,55,56]. Bacterial efflux pumps, proteins that remove toxic substances from cells, play a crucial role in MDR-TB.

As integral membrane proteins, efflux pumps orchestrate a complex resistance mechanism that covers different drug classes. The overexpression of efflux pumps such as Rv1258c (P55 efflux pump) enhances drug tolerance and potentially facilitates the acquisition of chromosomal mutations that confer increased resistance levels. This intricate process leverages the transmembrane electrochemical gradient of protons or sodium ions to expel drugs from the cell, effectively counteracting drug activity. PS5 actively extrudes and consequently confers resistance to multiple drugs, including rifampin. Efflux pumps, combined with permeability barriers, reduce the movement of antimicrobials through the outer bacterial membrane, adding another layer of complexity to the resistance mechanism [57,58]. While efflux pumps alone typically do not cause drug resistance, they contribute to other resistance mechanisms in *Mtb* and are crucial in causing elevated levels of resistance [59,60].

- Phenotypic Adaptation

The remarkable success of *Mtb* can be attributed to three key abilities, viz., the reprogramming of macrophages post-primary infection/phagocytosis to evade destruction, the initiation of well-organized granuloma formation, which includes various immune cells to establish a controlled environment for the host–pathogen interaction, and the ability to downregulate its central metabolism, halt replication, and enter a dormant state, making it exceptionally resilient against the defenses of the host and pharmacological treatment interventions. The mechanism of phenotypic adaptation enables bacteria to alter their physiological state when faced with challenging environmental conditions, particularly under antibiotic stress [61]. Metabolic reconfiguration of *Mtb* can significantly modify its metabolic state to endure demanding conditions. During exposure to drugs, the bacterium can enter a metabolically inactive state, which reduces susceptibility to the drug and leads to metabolic dormancy and establishes safeguarding conditions that restrict drug infiltration via biofilm development or exploration of alternative metabolic pathways that circumvent drug-targeted processes by leveraging metabolic heterogeneity. *Mtb* uses phenotypic adaptation as a vital survival strategy, enhancing its notable capacity to acquire and sustain multi-drug resistance [61].

- Oxidative Stress and Adaptation

Following *Mtb* invasion, the immune system of the host initiates its defense mechanisms against the pathogen, subsequently triggering the phagocyte process that produces reactive oxygen species (ROS) [62]. The host cells elevate the production of ROS to eliminate mycobacterial infection. However, the potential harm of overproduction of ROS cannot be overstated. It can harm host cells by exacerbating inflammation and related tissue damage, underscoring the urgency of research. For example, when the generation of ROS, such as hydrogen peroxide (H_2_O_2_) and superoxide anion, exceeds the necessary levels for cellular metabolism in the lungs, it can result in excessive tissue exposure to a persistent redox imbalance [63].

The role of oxidative stress induced by host macrophages is crucial in inhibiting the growth and development of *Mtb*. Certain drug-resistant strains of *Mtb* impede the redox defense of the host via different mechanisms. Mycolic acid, associated with the cell wall, is a physical barrier against host-related oxidative stress and can inhibit the oxidative stress response through its presence in the cell wall [64]. A point alteration in the *ndh* gene, responsible for encoding NADH II dehydrogenase in certain *Mtb* strains, leads to increased levels of NADH/NAD+, resulting in co-resistance to ETH and INH. These strains resist acidified nitrites and peroxides [65]. However, certain strains of *Mtb* possess a unique protein known as enhanced intracellular survival (Eis), which plays a fascinating role in identifying and countering ROS [66]. Certain strains of *Mtb* possess peroxiredoxin, including thioredoxin reductase (TrxR) and thioredoxin, which can mitigate and modify oxidative stress via disulfide reductase activity [67,68]. DosS and DosT act as redox sensors and activate the transcription factor DosR, which assists in the anaerobic survival of *Mtb* and contributes to the latent phase of infections [69,70]. In addition to causing tissue damage, ROS activates antimycobacterial agents such as INH and PA-824. *Mtb* develops resistance to this drug through inactivation of host-mediated oxidative stress, highlighting the ability of *Mtb* to manipulate the oxidative response of the host [71]. Oxidative stress is a significant underlying factor, and further research is urgently required to improve the different regimens for treating tuberculosis.

## 3. Advances in MDR-TB Treatment Strategies

### 3.1. Emerging Drug Regimens

#### Bedaquiline-Based Regimens

Bedaquiline (BDQ) or diarylquinoline TMC 207 represents the first adenosine triphosphate (ATP) synthase inhibitor targeting MRD-TB [72]. This drug represents a significant advancement in treating MRD-TB infections, with recent studies demonstrating remarkable efficacy against drug-resistant *Mtb* strains [73,74,75,76]. BDQ has a unique mechanism of action, which differs from existing anti-tuberculosis therapeutics as it explicitly targets mycobacterial ATP synthase (Figure 2), a crucial membrane-bound enzyme found in dormant and actively replicating mycobacteria. By binding to a particular site on subunit c, it disrupts the rotational movement of this subunit during catalysis or at the interface between the oligomeric subunit c and subunit a, thereby hindering the energy metabolism of the pathogen [77,78]. The substance has a remarkable capacity to eliminate mycobacteria across different microenvironments, which is partly attributed to its affinity for TMC207 under low pH and low proton motive force values [77]. The primary mechanisms conferring resistance to BDQ include mutations in the atpE gene, which encodes ATP synthase; alterations in the rv0678 gene, which regulates a drug efflux pump; and mutations in the pepQ gene (rv2535), which lead to low-level resistance to BDQ and CFZ [40].

It has been reported that BDQ can interact with the ε subunit [78,79]. BDQ has a significant impact on the immune system of the host. A recent study reported that BDQ improves the innate immune resistance of host macrophages against bacterial infections. Treatment with BDQ initiated a range of antimicrobial defense mechanisms, such as the fusion of phagosomes and lysosomes and autophagy. The effects observed were linked to the activation of transcription factor EB, which plays a role in the transcription of lysosomal genes, leading to improved intracellular killing of different bacterial species inherently resistant to BDQ. Notably, the role of BDQ as a host-directed therapy for different bacterial infections [80] underscores its potential and importance in pharmacology and immunology. It warrants additional exploration due to potentially significant and positive impacts when treating cancer patients with compromised immune systems [72].

The effectiveness of BDQ has been shown to reduce the treatment period for MDR-TB patients and improve the success rate of therapy [81,82,83,84]. BDQ is a fundamental element of the six-month treatment protocol for MDR/rifampicin-resistant(RR)-TB/BPaLM/BPaL, which includes bedaquiline, pretomanid, and linezolid, with or without moxifloxacin, and is the preferred regimen recommended by WHO for treating adolescents and adults > 14 years of age. BDQ is a key element of the nine-month all-oral regimen, recognized as the preferred treatment for eligible children and young adolescents < 14 years of age infected with MDR/RR-TB, as opposed to the more extended (18-month) regimens [83]. BDQ exhibits significant lipophilicity, characterized by an extended effective half-life, a complex distribution profile, and a slow elimination profile characterized by a gradual release from peripheral tissues [85]. BDQ is available as 400 mg tablets, administered daily for two weeks, which is then reduced to 200 mg three times a week for 22 weeks. BDQ must be taken with food, as this enhances bioavailability two-fold. BDQ undergoes metabolism via the cytochrome P450 isoenzyme 3A4 and is influenced by both inducers and inhibitors of this specific isoenzyme. Adverse drug reactions associated with BDQ typically affect the gastrointestinal, musculoskeletal, and central nervous systems. These include nausea, vomiting, diarrhea, abdominal discomfort, limb pain, joint pain, back pain, headache, and dizziness [86,87].

### 3.2. Precision Medicine in MDR-TB

For more than five decades, managing patients infected with TB has followed a standardized approach that overlooks the differences in human vulnerability to infection, immune response, pharmacokinetics, and the duration of treatment required to achieve a relapse-free cure. However, recent notable scientific discoveries and technological advancements offer a perspective for personalized rather than standardized management of patients with TB. This shift in approach can be used to optimize the selection of the most effective medications and host-directed therapies and tailor drug dosing and treatment durations, making it a topic of great interest for clinicians managing TB [88]. Precision medicine for tuberculosis encompasses tailor-made treatment regimens, therapeutic drug monitoring, and biomarker-guided therapy.

#### 3.2.1. Tailor-Made Treatment Regimens

Recent advancements in whole-genome sequencing are revolutionizing the application of genomics in the epidemiology and diagnosis of TB. The enhanced accuracy of innovative methods enables experts to pinpoint transmission with remarkable clarity and to develop customized approaches to decrease the occurrence of TB both locally and globally. Healthcare professionals, researchers, and policymakers are crucial in integrating these advancements into clinical practice. Furthermore, in contrast to the restricted commercially available molecular tests, diagnosing drug resistance via the complete genome allows for examining all drug resistance targets by using the catalog provided by the WHO. These advancements are progressively being integrated into clinical practice, ultimately facilitating the gradual control of TB and providing tailored care for every patient [89].

#### 3.2.2. Therapeutic Drug Monitoring (TDM)

Considering the complexities of managing MDR-TB, healthcare professionals can enhance the chances of achieving successful outcomes by implementing a TDM approach to therapy. TDM is a clinical approach that makes use of concentrations from the patient to modify therapy, enhancing the chances of achieving therapeutic drug levels while reducing the risk of toxicity [82]. The use of TDM to strengthen the management of TB has received strong support from established guidelines, including the ATS/CDC/ERS/IDSA clinical practice guideline for treating drug-resistant TB [90], and provides a solid foundation for implementation. Potential reasons for TDM may encompass compliance assessment, tailoring treatment, determining if a patient is receiving the correct doses, preventing toxicity related to drug concentration, and addressing issues related to drug–drug interactions [91]. It may be prudent to conduct TDM in all patients with MDR-TB instead of only using it when treating patients who exhibit a poor response to treatment [90].

#### 3.2.3. Biomarker-Guided Therapy

Without a timely indicator of treatment failure or relapses in patients infected with MDR-TB, biomarkers derived from host miRNA and *Mtb* RNA, assessed in extracellular vesicles (EV), provide a viable alternative for monitoring MDR-TB infections. The analysis of the payload of EV to determine differentially expressed miRNA before and after treatment, in addition to monitoring *Mtb*-derived RNA in serum EV from patients resistant to TB. A dual signal consisting of host-derived miR-let7e-5p and *Mtb*-derived RNA could indicate treatment failure or relapse after the treatment period has ended [92]. In numerous diseases, such as tuberculosis, biomarkers play a crucial role in diagnosing the condition, anticipating the emergence of an active phase, and assessing responses to treatment or vaccination [93], thereby keeping clinicians informed and aware of the progression of the disease.

### 3.3. Novel Drug Candidates

#### 3.3.1. Delamanid

Delamanid (DLM)/OPC67683 is an anti-TB agent from the nitro-dihydro-imidazooxazole class of compounds that inhibit mycolic acid synthesis in the bacterial cell wall. It is a potent weapon in the fight against drug-resistant tuberculosis, demonstrating its effectiveness when combined with other antibiotics [30,32]. DLM is a prodrug that must be activated by bioactivation before exerting antibacterial efficacy against both proliferating and dormant mycobacteria via the mycobacterial F420 coenzyme and the deazaflavin-dependent nitroreductase (rv3547) enzyme systems. Following stimulation, the synthesis of methoxy mycolic and ketomycolic acids is inhibited via the radical intermediate generated between DLM and the desnitroimidazooxazole derivative, resulting in the depletion of mycobacterial cell wall components and, ultimately, resulting in cell lysis [40,94]. Mutations in the genes associated with prodrug activation (fgd1 and ddn) and the F420 biosynthesis pathway (fbiA, fbiB, fbiC) have been characterized as resistance mutations. Phenotypic resistance to DLM has been documented in patients with multidrug-resistant tuberculosis who have not undergone prior treatment with DLM [40].

The recommended oral dose of DLM is 100 mg twice daily for people > 50 kg in weight and 50 mg twice daily for those weighing between 30 and 50 kg [40]. Healthcare professionals must consider the patient’s weight when prescribing DLM, as it can significantly affect the dose required and treatment outcome. DLM is poorly soluble in aqueous media, and absorption is enhanced two-fold when administered with meals. The absolute bioavailability is undetermined but ranges between 25% and approximately 47% [40]. DLM binds extensively to proteins with a capacity > 99%, which translates into a volume of distribution of 2100 L and a half-life ranging between 30 and 38 h. It has been suggested that albumin primarily facilitates the metabolism of DLM, supplemented by the involvement of P450 enzymes, particularly *CYP3A4* [94]. Gastrointestinal adverse effects are associated with DLM. DLM may induce QTc prolongation, an adverse effect linked to various medicines used to treat MDR-TB, including BDQ and fluoroquinolones [40].

#### 3.3.2. Pretomanid

Pretomanid, or PA-824, is the third medication to receive authorization from the Food and Drug Administration (FDA) in 2019, following BDQ and DLM [95]. It is an oral nitroimidazooxane that disrupts mycolic acid biosynthesis by obstructing hydroxy-mycolate oxidation to ketomycolate, thereby actively targeting replicating cells by disrupting cell wall synthesis. This agent demonstrates efficacy against non-replicating *Mtb* in anaerobic environments, acting as a respiratory toxin and inhibiting protein synthesis, which is attributed to the generation of intracellular nitric oxide [96,97,98]. However, the influence of nitric acid does not yield a notable bactericidal effect on bacteria that replicate aerobically [98,99]. PA-824 is a prodrug that requires metabolic stimulation through a deazaflavin (cofactor F420)-dependent nitroreductase (*ddn*) pathway [99]. Mutations in the genes linked to prodrug activation (fgd1 and ddn) and the F420 biosynthesis pathway (fbiA, fbiB, fbiC) have been described as resistance mutations to PA-824 [40]. The potential of PA-824 for treating extensively resistant tuberculosis (MDR-TB and XDR-TB) in combination with BDQ and linezolid as part of the BPaL course of therapy [95,100,101] brings hope and optimism to the field of infectious diseases.

The bioavailability of PA-824 at 50–1500 mg doses in humans was favorable and is significantly enhanced following a high-caloric fat meal relative to fasting conditions [102,103]. PA-824 has a half-life of 16 to 20 h and can be administered once daily. *CYP3A4* constituted around 20% of the metabolism in vitro [97], and everyday side events linked to PA-824 include peripheral neuropathy, acne, anemia, abdominal pain, nausea, vomiting, musculoskeletal pain, and headache [97].

### 3.4. Repurposed Drugs for MDR-TB

Patients encounter difficulties in adhering to prescribed regimens for the treatment of multi-drug-resistant tuberculosis due to considerable toxicity, low efficacy, and prolonged treatment durations leading to drug resistance (DR). The development of resistance to several first-line anti-TB medications requires the development of new TB therapies for treating drug-resistant individuals effectively. One key aspect of these new therapies is the need for a reduced treatment duration for drug-susceptible and resistant bacterial strains. However, establishing a new medicine regimen that integrates two or three innovative and effective pharmaceuticals is lengthy. It may take between 20 and 30 years with substantial financial investment, as observed with the development of BDQ and DLM. These challenges make medication repurposing a necessity, and the repurposing of previously approved pharmaceuticals for other conditions holds significant promise for the treatment of anti-DR-TB. Consequently, drug repurposing/repositioning is a fascinating field that involves discovering novel therapeutic applications for an existing medication, focusing on its pharmacodynamics and interactions with other receptors. This approach could improve the treatment of various diseases for which the medication was not initially authorized. These repurposed pharmaceuticals target several routes, reducing the likelihood of treatment resistance. Examples of these compounds include sulfonamides, sulfanilamide, sulfadiazine, clofazimine, linezolid, amoxicillin/clavulanic acid, carbapenems, metformin, verapamil, fluoroquinolones, statins, and NSAIDs. Their mechanisms of action are associated with immunomodulatory effects on the host, facilitating both host-directed and pathogen-targeted therapy options [104,105].

#### 3.4.1. Linezolid

Linezolid (LZD), an oxazolidinone derivative, is authorized to treat severe skin and soft tissue diseases, bacteremia, and pneumonia caused by Gram-positive bacteria. LZD eradicates *Mtb* by attaching to and obstructing tRNA in the peptidyltransferase center of the 50S ribosomal subunit, which comprises 5S rRNA and 23S rRNA [106,107]. Resistance arises through specific mutations that alter the ribosomal subunit within the drug-binding site [40]. Encouraging clinical evidence has supported the progressive repurposing of LZD for treating MDR and XDR-TB [107,108]. The standard oral treatment dose is 1200 mg, administered once, or 600 mg, administered twice daily. In the short term, headache, rash, and gastrointestinal side effects such as diarrhea and nausea are the most frequent adverse reactions [40]. Severe adverse effects resulting in treatment interruption with LZD occur in approximately 3–4% of patients undergoing short-duration treatment. Myelosuppression is a significant side effect of LZD, impacting approximately 28–33% of patients undergoing prolonged treatment with the drug [109]. Initial findings indicate that toxicity is dose-dependent and often manifests after a minimum of two weeks of treatment. Consequently, myelotoxicity is unlikely in patients receiving ≤ 600 mg daily dosages, even with a duration of therapy exceeding 20 months. This underscores the safety of using lower doses and should give healthcare professionals confidence when prescribing [110].

#### 3.4.2. Clofazimine

Clofazimine (CFZ) is predominantly utilized to treat leprosy. It is a lipophilic riminophenazine dye exhibiting both antimycobacterial and anti-inflammatory properties and was first identified as an anti-TB agent in the 1950s [40,111,112,113]. The precise mechanism of action of CFZ is still unknown; however, it seems to exhibit various effects on *Mtb*, such as disrupting redox cycling through the enzymatic reduction in CFZ by NDH-2, leading to the production of bactericidal ROS in addition to membrane destabilization and dysfunction by obstructing the electron transport chain in bacteria [113,114]. Evidence suggests that CFZ and BDQ affect the electron transport chain of *Mtb* [114]. The primary resistance mechanisms to CFZ are mutations in the rv0678 gene, which encodes a transcriptional repressor for the MmpL5 efflux pump, resulting in the overexpression of the multi-substrate efflux pump associated with drug resistance. These mutations also impart cross-resistance to BDQ. Notably, mutations in the pepQ gene (rv2535c) play a significant role in low-level resistance to BDQ, further highlighting the importance of this gene in drug resistance. Mutation in rv1979c is also thought to be associated with a transmembrane transporter exhibiting permease activity [40]. The typical oral dose of CFZ is 100 mg once daily, and the primary adverse effects of CFZ include skin pigmentation and gastrointestinal tract discomfort [40].

#### 3.4.3. Cycloserine

Cycloserine (CYS) exhibits a unique mechanism of action and has been used to treat MDR-TB since the 1950s [115]. It is a bacteriostatic agent indicated for incorporation and use in prolonged MDR-TB treatment regimens. Terizidone is a structural analog of CYS that is also effective for treating MDR-TB [40]. CYS is a cyclic counterpart of D-alanine and may inhibit alanine racemase and D-alanine ligase, thereby disrupting bacterial cell wall synthesis [40,116] without exhibiting cross-resistance with other anti-TB medications due to the distinct mechanism of action [117]. The processes underlying CYS resistance are intricate and involve genes associated with lipid metabolism, stress response, and transport systems [40]. However, the overexpression of the alanine racemase (alr) gene in *Mycobacterium smegmatis* is an essential and sufficient factor in imparting CYS resistance. A transversion from G to T in the alr promoter may result in the overexpression of this crucial gene, further highlighting its importance in the resistance mechanism [31]. With its unique mechanism of action and promising potential, CYS should intrigue healthcare professionals. In addition, its safety profile justifies its application in most instances, and it has been found to markedly enhance the likelihood of a positive result for patients with uncomplicated MDR-TB but not those who present with pre-XDR-TB or XDR-TB [116]. The maximum daily dose is 1000 mg, and the principal side effects linked to CYS include psychiatric conditions and central nervous system toxicity [40].

### 3.5. Host-Directed Therapies (HDT)

Adjunctive therapies, designed to ‘re-educate’ the immune system, offer a viable and alternative strategy to customize the response of the host to TB infection [118]. The proven effectiveness and attractiveness of HDT, given the significant influence of the immune response of the host on *Mtb* infection outcomes [119,120], offer potential opportunities to combat drug resistance. Enhancing the efficacy of tuberculosis therapy and disrupting the mechanism(s) essential for sustained persistence and replication while targeting routes affected by *Mtb* without directly engaging with it offers a strategic alternative to therapy [118,121,122]. As a result, HDT alleviates the burden of infection by functioning as an immunomodulator, thereby enabling the body to combat antibiotic-resistant pathogens while reducing the likelihood of developing resistance to susceptible new drugs, as *Mtb* cannot acquire resistance to a drug that targets host cell functions [104,123].

Host-directed immunomodulation by reducing treatment duration and preventing the emergence of resistance whilst increasing the susceptibility of *Mtb* to existing anti-tuberculosis medications and mitigating the response of the host inflammatory toxicity that compromises treatment efficacy will benefit patients [105,119] as the approach holds significant promise for the treatment of drug-resistant TB. Pharmaceutical compounds targeting the host cell rather than the *Mtb* bacillus are less susceptible to induced drug resistance, reducing selection pressure on the bacterium [122]. Furthermore, HDT offers a promising approach for the treatment of drug-resistant TB with minimal exposure to TB medications, thereby preventing or slowing the emergence and dissemination of superbugs. Combining HDT with conventional therapy facilitates synergism and the use of reduced doses, decreasing toxicity whilst maintaining treatment efficacy [104,121,122]. Combining HDT may also mitigate the hazards related to drug–drug interactions in patients with co-morbidities, such as those treated with antiretrovirals in TB-HIV-positive patients [124]. Some promising HDT candidates include corticosteroids such as prednisolone and dexamethasone, ibuprofen, aspirin [118,125,126], metformin [121,127], NSAIDs, vitamin A, zinc [118,128], and vitamin D_3_ [118,121].

Repurposed medications used to treat tuberculosis function as host-directed therapy, conditioning the immune cells of the host to accommodate the presence of tuberculosis, enhancing their antibacterial efficacy, and significantly reducing the duration required to eradicate the illness while minimizing inflammation and tissue damage [104]. This promising approach provides hope for the future of patient care. The primary mechanisms through which repurposed adjunctive compounds enhance tuberculosis treatment outcomes include modulation of inflammatory routes and pro-inflammatory mediators to attenuate inflammation and associated tissue pathology, thereby improving lung function and integrity; enhancement of the ability of the host immune response and reinforcement of immune and memory responses; augmentation of host bactericidal mechanisms; macrophage-mediated *Mtb* elimination in addition to a reduction in bacilli proliferation; and disruption and penetration of any granuloma to expose *Mtb* to anti-tuberculosis therapy [118].

Host-directed therapies, particularly those involving repurposed medications, are beneficial and essential for achieving the 2035 World Health Organization (WHO) End TB objectives [118], which underscores the urgency and importance of our collective efforts in this field of research.

#### 3.5.1. Metformin

Metformin is used to treat type 2 diabetes through mechanisms involving AMP-activated protein kinase (AMPK) dependence, independence, and Sirtuin I inhibition. These are crucial for detecting cellular energy levels and may facilitate the activation of autophagy to eliminate *Mtb,* mechanisms which have, in recent studies, been supported [71,104,105]. Metformin treatment augmented the protective immunological response and elevated ROS generation, inhibiting *Mtb* growth. The medicine demonstrated efficacy in eradicating medication-resistant bacterial strains by facilitating effective phagosome-lysosome fusion, alleviating chronic lung inflammation, augmenting the immune response, and boosting the effectiveness of conventional TB medications [129,130]. Metformin augments the release of IFN-γ from CD4+ and CD8+ T cells, modulates inflammation, and activates intracellular antimicrobial defenses [104,130,131]. Given the extensive utilization of metformin and current safety data, it is ideal as a primary choice for adjunct high-dose therapy when treating TB [71].

#### 3.5.2. Non-Steroidal Anti-Inflammatory Drugs (NSAID)

The host-directed therapeutic actions of NSAIDs are primarily influenced by their distinct pharmacokinetic features rather than alternative activity pathways, which result in various effects depending on tissue location and cell type. Ibuprofen has been extensively investigated for TB treatment, and aspirin has garnered interest in TB research [125]. The primary mechanism by which NSAIDs function in tuberculosis treatment is by alleviating inflammation resulting from the accumulation of monocytes, lymphocytes, and neutrophils [105]. NSAIDs demonstrate anti-inflammatory effects by inhibiting the cyclooxygenases COX-1 and COX-2 enzymes, which regulate pro-inflammatory and immunosuppressive mediators, including prostaglandins and leukotrienes. Inhibiting cyclooxygenase enzymes halts chronic inflammatory responses in the host, contributing to pathological lung lesions while enhancing bactericidal mechanisms and the immunological response to vaccines. The justification for using NSAIDs to treat HDT relates to suppressing pro-inflammatory COX enzymes, which mitigate excessive inflammation-related tissue damage and enhance host bactericidal function in persons with active tuberculosis [105,118].

#### 3.5.3. Vitamin D_3_

Many tuberculosis patients exhibit clinical vitamin deficiencies, including vitamin D_3_ [119]. Consequently, because of the association between vitamin D_3_ deficiency and susceptibility to tuberculosis, vitamin D_3_ has emerged as a significant focus of investigation for HDT-TB. Recent studies have highlighted the potential of vitamin D_3_ to improve the production of reactive oxygen and nitrogen intermediates, promote autophagy, and facilitate the production of antimicrobial peptides [71,121]. Vitamin D_3_ exerts an immunomodulatory action on the innate immune system by upregulating its response and inflammatory response, respectively, through its active form, 1,25-dihydroxy vitamin D_3_ (1,25D) pathway, leading to a reduction in the proliferation of *Mtb* in macrophages treated with 1,25D. Vitamin D significantly influences the innate immune system by promoting the expression of antimicrobial proteins and facilitating the formation of autophagosomes. Vitamin D also influences the adaptive immune system by promoting the formation of suppressive regulatory T cells and inhibiting the production of inflammatory Th17 cells. Consequently, the relationship between vitamin D and TB focuses mainly on enhancing bacterial removal via integrated innate and adaptive immune mechanisms and the reduction in tissue damage [121,132]. In addition to enhancing innate immune function through various mechanisms, such as the induction of autophagy, vitamin D plays a crucial role in modulating inflammatory responses, which is achieved by reducing the development of pro-inflammatory cytokines and chemokines, raising the levels of anti-inflammatory cytokines, and influencing the T-cell response [120].

### 3.6. Enhancing Autophagy in Therapy

#### Autophagy Inducers

The activation of autophagy in diseased cells by diverse autophagy-inducing compounds (AIC) has emerged as a promising alternative treatment strategy for tuberculosis [121]. Autophagy is a catabolic mechanism that facilitates the lysosomal breakdown of cellular constituents, including invading pathogens such as *Mtb,* to maintain cellular homeostasis [133,134]. Macrophages exhibit robust antimicrobial responses to *Mtb* infection via autophagy. Nonetheless, *Mtb* has developed advanced approaches to evade, disrupt, and manipulate the antimicrobial functions of macrophages by disrupting the production of protective Th1-type cytokine, vacuolar membrane trafficking, or autophagy activation for extended survival rather than merely eliminating the host [135]. Autophagy is vital in sustaining intracellular balance and essential to the immune response. Therefore, modulators of autophagy or activating this process through different drugs or agents is a promising opportunity in HDT against *Mtb* infection, whether used alone or in combination with standard treatments, including for drug-resistant strains. The modulation of autophagy activation plays a crucial role in managing inflammation, enhancing the effectiveness of both innate and adaptive immune responses against *Mtb* [134,136,137]. Key factors that facilitate the activation of autophagy encompass vitamin D receptor signaling, the AMP-activated protein kinase pathway, Sirtuin 1 activation, and nuclear receptors [134].

Innovative approaches in anti-TB treatment have been proposed by manipulating autophagy activation, including using surface-functionalized or modified nanoparticles (NPs) that encapsulate traditional anti-TB medications and other AIC designed for HDT [121]. NP augments the efficacy of AIC, thereby enhancing stability, facilitating cell targeting, and creating avenues for multimodal treatment [137]. The established HDT drugs are crucial in pathological inflammation, phagolysosomal fusion, lysosomal functions, and the antimicrobial response of host cells infected by *Mtb*. While agents that activate autophagy could serve as potential therapeutic options for HDT-TB, numerous other biological pathways, including autophagy, play a role in the ability of the host to defend itself against TB infection. For instance, vitamin D is often used for treating HDT-TB and was used before the advent of antibiotics. Beyond a capacity to activate autophagy, vitamin D exhibits a range of activity on diseased cells and tissues, including a significant role in enhancing direct antimicrobial defense through cathelicidin and modulating inflammation [134].

A limitation in the field pertains to clinical usage, specifically regarding the potential for targeted drug administration to disease sites and the possibility that autophagy-adjunctive therapies might shorten the period of antibiotic treatment. Ongoing and forthcoming investigations into autophagy-based HDT therapeutic candidates will enhance our understanding of the antibacterial role of autophagy. Nonetheless, this approach might also play a role in advancing therapeutic strategies for TB [134].

## 4. Innovative Drug Delivery Systems

### 4.1. Nanoparticle-Based Drug Delivery

The primary challenges related to conventional tuberculosis medications include inadequate aqueous solubility, limited penetrability, systemic toxicity at therapeutic doses, off-target accumulation, bacterial mutation resulting in multidrug-resistant strains, and diminished bactericidal efficacy against bacteria within macrophages or infected deep tissues. In this respect, nanomedicine has been recognized as providing unique benefits that may effectively tackle the previously reported challenges concerning treating TB infection [138]. Nanoparticles serve as drug nanocarriers or nanocontainers (Table 3) with the potential to augment therapeutic efficacy and enhance patient adherence to tuberculosis treatment, leading to more optimistic treatment outcomes. They offer advantages such as substantial and multifaceted drug encapsulation, improved immune response, significant passive permeability of the payload, sustained release, facilitation of autophagy-inducing activity, reduction in administered doses, precise delivery, decreased dosing frequency, minimal adverse effects, and the utilization of multiple synergistic mechanisms to enhance antimicrobial activity and counteract antibiotic resistance [28,121,137,139,140].

The mechanisms of antimicrobial activity when delivered from nanoparticles include alteration of essential proteins, inhibition of enzyme activity and protein synthesis, incorporation into DNA bases, generation of oxidative stress, disruption of cell signaling, inhibition of biofilm formation, penetration of cell membranes, and inhibition of cell wall synthesis [31]. The modulation of nanocarrier attributes, including surface composition, charge, shape, temperature, redox state, particle size, pH, hydrophobicity, hypoxia, and Zeta potential, may influence drug uptake by alveolar macrophages [26,140]. An alternative method involves targeting ligands on the nanocarrier that engage with specific receptors on macrophages, which is referred to as active or ligand-mediated targeting. Nanoparticles (NPs) can activate macrophages, driving them into a bactericidal state that effectively targets and eliminates intracellular *Mtb*. The surface of the nanoparticles can be modified with ligands to engage with particular macrophage surface receptors that play a role in macrophage activation [154]. Approaches independent of specific ligands are termed enhanced permeability and retention effects or passive targeting. Carriers based on polymers and polysaccharides, liposomes, and metallic nanoparticles have gained interest in the active and passive targeting of antimicrobial agents in MDR-TB medication administration. Their potential to improve drug solubility, stability, and bioavailability offers hope for the future of tuberculosis treatment. Furthermore, facilitating and regulating the release and targeted medication administration to the infection site is possible [139,157].

#### 4.1.1. Liposomal Systems

Liposomes are remarkable spherical vesicles comprising lipids, such as phospholipid and cholesterol, to enhance drug delivery and mitigation of drugs. They can encapsulate hydrophobic pharmaceuticals within the hydrophobic core of their bilayers and water-soluble substances in the hydrophilic center region, thereby facilitating their transport across biological barriers. These are the well-studied systems for the controlled administration of medications to the lungs, as they can be formulated with phospholipids naturally present in the lungs as surfactants. The drug delivery mechanism requires either fusion with the cell membranes for drug release or endocytosis, which entails entering mononuclear phagocytic macrophages. They can enhance the pharmacokinetics of medicines and mitigate toxicity by lowering systemic exposure to elevated drug concentrations [28,140].

Liposomes were successfully synthesized with dipalmitoylphosphatidylcholine and cholesterol as a carrier for Zn(II) phthalocyanine (ZnPc), a non-toxic photosensitizer for the inactivation of susceptible (ATCC 27294) *Mtb* and MDR-TB (9037R) [140]. The ZnPc-loaded liposomal formulation, when compared with the unmedicated or control liposomes, successfully inactivated the two pathogen strains used in this investigation, providing a significant breakthrough in the field. The duration of incubation and light exposure influenced the photoinactivation process. A substantial reduction in three (3) log10 CFU/mL following two (2) hours of incubation with 75 J/cm^2^ or 150 J/cm^2^ irradiation of the susceptible strain was observed. For MDR-TB, it was necessary to modify the incorporation time to four (4) hours and increase the light exposure to reduce three (3) log10 CFU/mL. Consequently, applying photodynamic antimicrobial chemotherapy with ZnPc-liposomes presents a potential alternative for treating MDR-TB, demonstrating an impressive reduction of 99.9% in mortality in vitro [140].

Niosomes resemble liposomes but include a surfactant bilayer with external and internal hydrophilic termini bared to the aqueous phase. At the same time, the hydrophobic chains are oriented towards one another inside the surfactant bilayer [158]. Ethionamide (ETH) is a second-line anti-TB agent, making it a preferable treatment option for MDR-TB. However, it is associated with transient, asymptomatic increases in serum aminotransferase levels and, in rare cases, can lead to severe acute liver injury [159]. The negative impact of ethionamide prompted Sadhu et al. [141] to develop niosomes to encapsulate the drug. This approach aims to enhance the therapeutic efficacy of the pharmaceutical by prolonging its presence in the bloodstream, using these vesicles as a reservoir for controlled drug release, addressing drug resistance challenges, shortening the duration of treatment, and minimizing drug–drug interactions, resulting in better patient adherence and treatment outcomes whilst minimizing drug-related toxicity. ETH-loaded niosomes prepared by thin film hydration exhibited sustained release of 94.89% of the payload over 24 h, implying a potential reduction in dosing frequency [141].

The encapsulation of ETH within niosomes prepared through thin-film hydration led to regulated drug release, enhanced efficacy, and improved safety compared with the unencapsulated drug [142]. The formulation effectively increased drug delivery to the lungs of mice over an extended duration, resulting in reduced bacterial counts in lung homogenates [142].

A long-acting dual drug-loaded self-assembling niosome technology incorporating lipophilic ETH and hydrophilic D-Cycloserine to treat MDR-TB effectively has been reported [143]. A Box–Behnken experimental design was used to develop and optimize the niosomes, and the formulation demonstrated commendable stability over 6 months. Hemodialysis studies revealed that administration of the dual drug-loaded niosomes via the intravenous route was safe, and the MIC for the niosome technology was the lowest compared with free drug and single drug-loaded niosomes. The retarded rate of release of ETH and rapid initial release of D-Cycloserine played a significant role in the efficacy. The combined effect of the two drugs in the niosomes demonstrated a more effective treatment option for tuberculosis when compared with the pure drug combination [143].

Notwithstanding the numerous advantages of liposomes, including their safety and biocompatibility profiles, their principal drawback as nanocarriers is their instability in plasma. Selective serum proteins such as opsonins adhere to the surfaces of liposomes upon entry into the systemic circulation and alert the mononuclear phagocyte system (MPS) to their presence, resulting in their removal from the bloodstream. Nonetheless, opsonization and removal can be mitigated through functionalization with polyethylene glycol (PEG) or other ligands, including antibodies, that enhance precision targeting of the infected location [160].

#### 4.1.2. Metallic Nanoparticles

Metal nanoparticles (MNP) have attracted considerable interest due to their medicinal applications, especially in antibacterial, drug delivery, and theragnostic applications. These nanoparticles exhibit significant antibacterial efficacy and biocompatibility, rendering them promising agents in the fight against antimicrobial resistance. Different MNPs, including iron oxide, zinc oxide, silver, and gold, have demonstrated promise in boosting the efficacy of medicine against resistant microorganisms. MNP can disrupt bacterial cell membranes and produce ROS, including superoxide anions, hydrogen peroxide, and hydroxyl radicals, which disrupt DNA replication and amino acid synthesis, thereby compromising microbial cell membranes. This disruption makes the development of bacterial resistance unlikely, providing reassurance about the effectiveness of MNP in combating antimicrobial resistance. The positively charged magnetic nanoparticles may engage with the negatively charged bacteria, resulting in lipid oxidation and cell death. Moreover, MNP can eliminate microbes by releasing ions [31,157,161]. The mechanism by which MNPs exert their effect on bacteria is intricate. MNP can simultaneously target multiple cellular structures, complicating the development of adaptive responses and consequently reducing the likelihood of developing bacterial resistance. MNP also serves as a unique delivery system for antibacterial drugs, protecting them against enzymatic and other degradation pathways. Their distinctive characteristics, including an ability to enhance delivery with greater specificity and diminished adverse effects, make them a fascinating area of research and development [31,157,161].

The efficacy of rifampin (RIF) when treating MDR-TB has been enhanced [144] using engineered polydopamine-coated silver nanoparticles (Ag-PDA NP) and revealed that minimum inhibitory concentration (MIC) tests performed with different ratios exhibited a synergistic interaction between Ag-PDA NP and RF, with the most effective antimycobacterial outcome against the multidrug-resistant strain of *Mtb* occurring at a mass proportion of 2 Ag-PDA NP to 8 RF. The synthesized and characterized RF-loaded Ag-PDA nanoparticles show that this drug-loaded metallic nano-formulation is not just a potential solution but a promising one to limit the growth of multidrug-resistant strains of Mycobacterium and maintain the efficacy of RIF in clinical applications [144].

The anti-tubercular properties of silver nanoparticles, when used in combination with anti-tuberculosis medications, have been reported and involved in vitro experiments in which 65 white mice were used with a model of resistant TB [145]. The apparent anti-tubercular effects of the nanocomposite were demonstrated using silver nanoparticles and isoniazid.

A 2019 study reported the antimycobacterial efficacy of combination treatment with transition metals and antibiotics against *Mtb* strains resistant to first-line drugs [146]. The findings suggest that a combination of INH and AgNO_3_ exhibited a synergistic effect, demonstrating bactericidal activity against a clinical strain of *Mtb* resistant to isoniazid [146].

Silver nanoparticles (AgNP) and zinc nanoparticles (ZnNP) exhibited effectiveness against *Mtb* and an MDR strain, with a MIC of 1.25 mg/mL [147]. The AgNP demonstrated superior antimicrobial efficacy when compared with ZnNP. The effectiveness of these nanoparticles in combating drug-resistant pathogens positions them as a promising option for therapeutic application [147].

A study showcasing the potent antimycobacterial properties of AgNP was reported in 2018 and focused on reference strains of *Mycobacterium bovis* and *Mtb* H37Rv, in addition to an MDR strain of *Mtb* and clinical isolates of both *M. bovis* and *Mtb* to determine the MIC of AgNP using the microplate Alamar blue assay. This study brings hope and optimism as it reveals the potential of AgNP as a promising chemotherapeutic agent against Mycobacterium *species* [148].

The exceptional potential of mixed metallic nanoparticles against MDR-TB has sparked interest in their application [147]. The anti-tubercular efficacy of metallic nanoparticles, specifically magnesium oxide nanoparticles (MgONP) and zinc oxide nanoparticles (ZnONP), concerning MDR-TB, remains unexamined. A study was initiated to compare the effects of different doses of a blend of MgONP and ZnONP on two clinical isolates, viz., MDR-*Mtb* and reference strain [149]. The research assessed the MIC and minimum bactericidal concentrations (MBC) of the ZnONP, MgONP, and MgONP-ZnONP against H37Rv *Mtb* and MDR-*Mtb*. The ZnONP and MgONP-ZnONP exhibited bactericidal properties and synergistic benefits against MDR-TB [149].

The antimycobacterial properties of Ag, ZnO, and Ag-ZnO NP against MDR and XDR-*Mtb* have been reported [150]. The MIC results demonstrated the inhibitory effects of these nanoparticles on these two strains of *Mtb*. Nonetheless, MBC results showed that Ag, ZnO, and Ag-ZnO NP, whether used individually or in combination, were ineffective in eradicating MDR- or XDR-*Mtb*. These nanoparticles are promising antimycobacterial nanodrugs due to their bacteriostatic activities against drug-resistant strains of *Mtb*. However, additional research is necessary to validate the bactericidal properties of these nanoparticles against TB [150].

Nanoparticles, with bactericidal and immuno-potentiating attributes, are currently being explored as an antibiotic alternative for their potential to reduce antibiotic doses, minimize toxicity, and reduce the likelihood of multi-drug resistance [31]. This reduction in toxicity offers a reassuring prospect for the safety of future therapies. However, further research on the synergistic effects of metallic nanoparticles with traditional anti-TB medications and novel anti-tubercular medicines is required. This research could significantly enhance the antibacterial efficacy of isoniazid and rifampicin against MDR-TB, which has acquired resistance.

#### 4.1.3. Polymeric Nanoparticles (PNP)

PNP nano-systems can enhance the efficacy of chemotherapeutic agents and mitigate the adverse reactions of anti-TB medications through the encapsulation and conjugation of therapeutic agents in carrier technology. The nano-systems can be manufactured from many natural or synthetic precursors, including collagen, chitosan, gelatin, albumin, polyethylene glycol, polylactic acid, poly (lactic-co-glycolic acid) (PLGA), and polycaprolactone (PCL). PLGA copolymers are extensively used for the delivery of anti-TB medicines. Moreover, polymers used to manufacture PNP can be transformed into different forms, such as micelles, vesicles, dendrimers, or hybrid inorganic-polymer nanocarriers [160,162]. Polymeric nanoparticles have generated significant interest as promising antibacterial drug delivery systems as they exhibit numerous advantages, including effective cargo dissolution, entrapment, encapsulation, surface bonding and/or functionalization, antibiotic properties, the capability to form antimicrobial groups for targeted destruction, biocompatibility, biodegradability, co-delivery of diverse drugs, accumulation on cell membranes, low toxicity, stability, and potential synergistic therapeutic activity [160,162,163,164].

Due to the toxicity, cost, and limited effectiveness of drugs used to treat MDR-TB, a nano-formulation was used clinically to deliver moxifloxacin (MOX), econazole (ECZ), and ethionamide (ETH) loaded into PLGA nanoparticles, and their therapeutic efficacy was assessed in an animal model infected with MDR-TB. The treatment of MDR-TB-infected mice with weekly doses of a three-component nano-formulation viz., PLGA-NP-ECZ + PLGA-NP-MOX + PLGA-NP-ETH, resulted in the elimination of bacilli from both the lungs and the spleen of the mice [151]. This investigation represents the initial documentation regarding the possible effectiveness of a mixture of ECZ, MOX, and ETH NP in treating MDR-TB, with promising potential for clinical application [151].

The high intracellular drug concentration resulting when using PNP and their ready internalization into macrophages led to the development of alginate-modified PLGA nanoparticles loaded with the hydrophilic compounds amikacin and moxifloxacin and two water–oil–water (w/o/w) emulsion methods for the targeted therapy of MDR-TB [152]. The antibacterial efficacy of the resultant PLGA NP in *Mtb*-infected macrophages was investigated. In the untreated group, the dual-encapsulated NP exhibited a bacterial viability of 0.6%. In contrast, the nanoparticles containing amikacin and moxifloxacin exhibited viability of 6.49% and 3.27%, respectively, thereby indicating that the synergistic effect of amikacin and moxifloxacin in the PLGA NP resulted in more significant inhibition of the viable bacteria. The alginate-encapsulated PLGA nanoparticles loaded with amikacin and moxifloxacin can be used to reduce the dosage of these medications, enhancing patient adherence to therapy and potentially mitigating undesirable side effects associated with higher doses; however, additional in vivo investigations are necessary to validate this potential [152].

Studies have demonstrated the safety and efficacy of sonodynamic antibacterial chemotherapy (SACT) as a potential solution to the rising threat of drug-resistant bacteria. By using sonosensitive-loaded nanoparticles with targeted therapeutic capabilities, SACT could effectively eradicate bacteria without the risk of precipitating drug resistance [165,166]. In response to the challenge of MDR-TB, this concept was used to develop levofloxacin-loaded PLGA-PEG NP conjugated with the BM2 aptamer on their surface using1-ethyl-3-(3-dimethylamino propyl) carbodiimide (EDC) and N-hydroxysuccinimide (NHS) as cross-linking agents to prepare BM2-LVFX-NP [153]. The nanoparticles were designed to investigate antimycobacterial efficacy and the fundamental mechanism of activity of the levofloxacin-loaded nano-sonosensitizer with specific therapeutic action against Bacillus Calmette-Guérin bacteria (BCG) used as a model for *Mtb*. The study revealed a significant production of ROS during treatment with BM2-LVFX-NP using ultrasonic activation. Both in vitro and in vivo studies demonstrated the robust targeting specificity of BM2-LVFX-NP for BCG, and the BM2-LVFX-NP-mediated SACT exhibited potent antibacterial efficacy against BCG and suppressed the proliferation of subcutaneous abscesses without any discernible adverse reactions. Consequently, an ultrasonic-activated therapeutic nano-platform incorporating an aptamer-specific moiety demonstrates significant capability as a practical approach for additive targeted treatment against *Mtb* infections, ensuring biosafety [153].

The polysaccharide 1,3-β-glucan is recognized for activating macrophages and producing pro-inflammatory signals, including ROS and nitrogen species (ROS). β-glucans interact with Dectin-1 located on the surfaces of macrophages, activating multiple downstream signaling pathways that enhance pro-inflammatory gene expression and the production of intracellular ROS/RNS. Pro-inflammatory cytokines generated via Dectin-1 activation encompass IL-12 and TNF-α [167], vital for managing *Mtb* infections [168]. Curdlan-functionalized PLGA NP designed for HDT exhibited enhanced and expedited release of the pro-inflammatory cytokine TNF-α in macrophages, reducing intracellular *Mtb* amount inside these cells [154]. This investigation further demonstrated that curdlan exhibits a strong binding affinity for the Dectin-1 receptor, facilitating targeted delivery of anti-TB drugs and AIC to macrophages. This mechanism enhances pharmaceutical activity in specific cells, simultaneously reducing immunotoxicity. The findings indicate that the nanocarrier exhibited bio-safety properties, highlighting the need for further investigation into its potential as a host-directed therapeutic approach for intracellular activity against viable *Mtb* [154] and MDR-TB.

Macrophages have mannose receptors on their surfaces that recognize and bind to non-reducing terminals of mannose moieties, thereby promoting the cellular uptake of nanoparticles. Consequently, using mannosylated nano-formulations may be a promising approach for targeting alveolar macrophages for the delivery of active therapeutics to treat MDR-TB. Incorporating mannose into a drug delivery vehicle that exhibits enhanced uptake of carriers by macrophages could result in improved efficacy and reduced side effects. This phenomenon may be linked to the preferential absorption of mannose by alveolar macrophages, facilitating the targeted accumulation of drug-loaded nanoparticles at the desired sites of action [169,170].

Mannose receptor-targeted bioadhesive chitosan NP encapsulating clofazimine to treat drug-resistant TB has been reported [155]. In vitro drug release at pH 7.4 was gradual and prolonged, and uptake tests in C2C12 cell lines demonstrated that the mannosylated nanoparticles were more efficiently absorbed than non-targeted and conventional medicines. The luciferase reporter phage (LRP) was tested against the H37Rv strain and revealed that clofazimine NP exhibited 49.5 times greater inhibition and antimycobacterial efficacy compared with pure clofazimine alone, reassuring the potential of this combination in tuberculosis treatment. This remarkable activity may be ascribed to improved drug distribution due to the favorable bioadhesive characteristics of the chitosan-based nanoparticles. Following regulatory validation, these nanoparticles may be clinically relevant to target macrophages infected with Mycobacterium and treat drug-resistant tuberculosis [155].

Innovative combinations of drugs and advanced targeted drug delivery technologies may enhance the management of anti-TB drug resistance significantly. Research indicates that fluoxetine, a serotonin reuptake inhibitor, may be beneficial in treating infectious diseases caused by mycobacteria [171,172]. A nano-system containing isoniazid and fluoxetine-conjugated multi-walled carbon nanotube nanofluid was developed to enhance drug delivery efficiency and address resistance in vitro [156]. It was established that fluoxetine exhibited a synergistic effect when combined with isoniazid. This suggests that their combined application in treatment regimens may significantly impact the management of infections caused by all clinical strains of *Mtb*. The expression of isoniazid resistance genes, such as *inhA* and *katG*, along with the secretion of cytokines TNFα and IL6, is effectively regulated by this drug delivery system [156]. These findings corroborate those reported in earlier studies, indicating that serotonin receptor agonists or antagonists can stimulate the autophagy pathway and promote the elimination of TB mycobacteria. In conjunction with host-targeted molecules, this nano-drug delivery approach can advance the production of a new generation of antibiotics characterized by high therapeutic efficacy, reduced side effects, and an ability to address drug resistance challenges; however, further comprehensive studies on this nano-formulation pertinent to signaling pathways are required [156].

Notwithstanding recent progress in developing PLGA NP DDS for treating TB, numerous challenges persist, requiring urgent resolution. The lack of specific regulatory criteria for these studies’ characterization, research design, and statistical analysis is a significant obstacle to the clinical translation of nano-formulation use. Enhancing collaborative practices is crucial for translating nanotechnology from successful proof of concept experimentally to clinical use, and supplementary in vivo data are necessary. Some studies suggest that the PLGA NP medication delivery technology exhibits efficacy in preclinical models of infectious TB. However, this is insufficient, as upcoming clinical trials will depend on available preclinical evidence. Investigations into PLGA nano DDS for TB therapy are still in their infancy, and additional funding is required to develop the capability to produce commercially viable micro/nano-formulations [173].

### 4.2. Gene Therapy and RNA-Based Therapy in the Treatment of MDR-TB

TB, particularly the MDR-TB forms, poses a significant global health challenge requiring therapeutic strategies. These strategies include gene therapy and non-coding RNA, such as small interfering RNA (siRNA), microRNA (mRNA), long non-coding RNA (lncRNA), and RNA interference (RNAi), which have emerged as promising avenues to combat the challenge. These technologies facilitate targeting the *Mtb* pathogen and host cellular mechanisms to enhance therapeutic efficacy.

#### 4.2.1. Gene Therapy

Gene therapy involves the introduction, removal, or alteration of genetic material within the cells of patients to treat diseases. In MDR-TB, gene therapy strategies aim to enhance the host’s immune response or target *Mtb* directly. The CRISPR-Cas9 system enables precise editing of genetic material and has been applied to study *Mtb* pathogenesis and drug resistance mechanisms. Researchers can identify novel drug targets and understand bacterial survival strategies by knocking out or modifying specific genes in the *Mtb*. CRISPR-based diagnostics have been developed to rapidly detect drug-resistant TB strains, facilitating timely and appropriate treatment interventions. While direct therapeutic applications of CRISPR in TB treatment are still in the early stages of development and use, the technology holds promise for developing targeted antimicrobial agents and enhancing our understanding of the biology of *Mtb* species [174]. Examples of the proposed application of the CRISPR system in MDR-TB treatment include reprogramming the endogenous CRISPR system of *Mtb*. Researchers have harnessed the endogenous type III-A CRISPR/Cas10 system of *Mtb* for efficient gene editing and RNA interference by transforming a mini-CRISPR array plasmid and avoiding the introduction of exogenous proteins whilst minimizing proteotoxicity. This system has been applied to single- and multiple-gene RNAi and genome-wide RNAi screening to identify *Mtb* genes regulating in vitro and intracellular growth [175]. A CRISPR-guided mutagenic DNA polymerase system has been developed in fast-growing *Mycobacterium smegmatis* and slow-growing *Mtb*. This system combines a Cas9 nickase with an error-prone DNA polymerase to introduce random substitution mutations within target gene(s). It has been used to detect novel resistant mutant organisms, potentially aiding in identifying drug-resistant mutations of *Mtb* [176]. The finding of low-abundance sequences by hybridization (FLASH-TB) diagnostic tool techniques has been applied to detect antibiotic-resistant mutations in *Mtb*. This method uses CRISPR to amplify candidate genes linked to resistance against first- and second-line drugs, facilitating swift and precise identification of drug-resistant TB strains [177].

Furthermore, researchers have explored gene therapy to boost the immune defense of the host organisms against *Mtb*. For example, enhancing the expression of cytokines such as interferon-gamma (IFN-γ) has been investigated to improve macrophage activation and mycobacterial clearance [178], and gene editing tools like CRISPR-Cas9 have been proposed to disrupt essential genes in *Mtb*, inhibiting survival and replication [179]. While direct application in clinical settings is still under investigation, this approach holds potential for future therapeutic developments.

A study by Rahman et al. reprogrammed the endogenous type III-A CRISPR system of *Mtb* to create a versatile tool for effective gene editing and RNA interference, demonstrating its potential for robust gene knock-in/knockout processes and genome-wide RNAi screening [175]. In addition, CRISPR-guided mutagenesis and gene editing have been proposed as strategies to combat drug-resistant strains of *Mtb*, offering new avenues for tuberculosis research and drug development [180]. CRISPR screening has been used for genetic interaction mapping of *Mtb*, providing insight into gene function and identification of potential therapeutic targets [181]. Other gene therapy strategies have been investigated for treating *Mtb* and MDR-TB. One notable example involved using adenoviral vectors to deliver therapeutic genes that enhance the host’s immune response against *Mtb*. An adenoviral vector encoding for osteopontin (AdOPN) was administered to *Mtb*-infected mice and led to heightened immune responses and improved control of bacterial load in the lungs, suggesting that AdOPN may be a potential adjunct to traditional chemotherapy [182]. Another approach focuses on HDTs that modulate the immune system of the host to combat *Mtb* infection. Gene therapy techniques have been used to alter the expression of specific host genes involved in immune regulation, thereby enhancing the ability of the body to control or eliminate the pathogen. The modulation of gene expression of genes encoding for cytokines or other immune mediators through gene therapy has shown promise in preclinical models [183] and highlights the potential of gene therapy approaches beyond CRISPR-Cas9 to treat *Mtb* and MDR-TB, offering alternative strategies to enhance host immunity and improve disease outcomes.

#### 4.2.2. RNA-Based Therapy for Treatment of MDR-TB

Research into RNA-based therapies for TB, including MDR-TB, has focused on using RNA interference (RNAi) mechanisms with small interfering RNA (siRNA) and microRNA (miRNA) molecules to modulate the immune response(s) in the host and target *Mtb.* For example, the inhibition of miR-27a was reported to enhance the ability of the host to control *Mtb* infections in mice, and the miR-27a antagomir reduced bacterial loads and diminished lung pathology, indicating that inhibition of miR-27a could be a promising therapeutic strategy for treating TB [184]. The miRNA miR-let-7e-5p has been identified as a potential biomarker for monitoring MDR-TB treatment, and variations in levels of expression align with the different phases of treatment, supporting its use for evaluating therapeutic efficacy [92]. Another miRNA, miR-155, is upregulated during *Mtb* infection, enhancing the antimicrobial activity of macrophages through an increased generation of pro-inflammatory cytokines. Modulating miRNA compounds such as miR-155 may strengthen the defenses of hosts against MDR-TB [185]. Differentially expressed lncRNA in peripheral blood mononuclear cells (PBMC) of MDR-TB patients have been proposed as potential biomarkers and therapeutic targets [186]. Specific lncRNAs in PBMC have been identified in patients with active TB, further underscoring their potential utility as biomarkers for diagnosing and monitoring treatment in patients with TB [187]. These findings collectively suggest that targeting non-coding RNA, including miRNA and lncRNA, may be significant for advancing RNA-based therapies in treating MDR-TB.

Despite their therapeutic potential, both RNA-based and gene therapy approaches for treating MDR-TB pose significant challenges that limit their clinical application. A major hurdle is the delivery of the therapeutic agents to infected cells while avoiding off-target effects, which may lead to unintended gene silencing, toxicity, or genetic modification [188]. Drug delivery systems must shield RNA molecules from degradation by nucleases and ensure precise targeting. However, these systems often raise safety concerns, such as immune responses triggered by viral vectors used in gene therapy [189]. Furthermore, ncRNA and gene therapy are complex due to interactions in the biological system. A single molecule may regulate multiple genes for ncRNA, making it difficult to predict and control exact therapeutic outcomes [190]. Gene therapy carries the risk of insertional mutagenesis when therapeutic genes integrate into the host genome [191]. The cost and complexity of producing these therapies with high purity and functionality further limit their scalability and accessibility, particularly in TB-endemic regions. The absence of thorough research on long-term safety and efficacy for both approaches is a significant barrier to clinical adoption [192]. Addressing these challenges requires innovative stabilization, delivery, and specificity technologies, and rigorous preclinical and clinical investigation. Furthermore, vector design and regulatory framework advancements are critical to making these cutting-edge therapies viable options for combating MDR-TB.

### 4.3. Pulmonary Delivery Systems

Transforming current anti-TB medications into inhalable nanoparticulate systems presents a potential approach to address the difficulties linked with oral treatment, as these systems may improve drug retention in pulmonary tissues and maintain therapeutic concentrations in the diseased lung(s) [193]. Since *Mtb* targets the lungs, administering drugs via the pulmonary route helps promote efficient alveolar transcytosis. This approach circumvents hepatic first-pass metabolism and, due to the extensive vascular network, enhances the delivery of therapeutic agents, potentially increasing drug concentrations at the site of infection, which may improve their effectiveness [194]. Interestingly, administering lower doses of TB medications via inhalation can still yield effective treatment outcomes, thereby minimizing the risk of toxicity while improving localized drug concentration [195]. Aerosolized formulations and inhalation devices, including nebulizers, dry powder inhalers (DPI), soft mist inhalers (SMI), and metered-dose inhalers (MDI), represent advancements in pulmonary DDS designed to deliver anti-TB medications directly to the lungs. This approach increases local drug concentrations at the site of action while minimizing systemic side effects. Nevertheless, dry powder inhalers (DPI) have significant benefits owing to their stability compared with liquid or suspension-based formulations and their capacity to administer a high dose of drugs directly to the lungs [194,196,197].

Concerns regarding the administration of anti-TB drugs via the oral route in tablets or capsules and the parenteral route include potential drug–drug interactions, a slower onset of action, and the possibility of degradation in the gastrointestinal environment. In addition, subtherapeutic drug concentrations in the lungs due to hepatic first-pass metabolism may develop resistant *Mtb* strains and require elevated doses to achieve high concentrations at the site of action, and insufficient drugs may penetrate necrotic lesions. Other considerations include excessive peripheral neuropathy, serious side effects, and low tolerability of intravenous formulations. However, the rationale for inhalation delivery includes low systemic exposure of the drugs, high concentration of drugs at the site of action, which can potentially reduce the frequency of dosing requirements for successful therapy, avoid potential pharmacokinetic drug–drug interactions, food–drug interactions, high penetration of the drugs in necrotic lesions of the lungs, early sterilization of the sputum, or early bactericidal activity, all of which may increase patient adherence [194,197].

Numerous antimicrobial drugs have received approval or are in preclinical investigation or clinical trials to evaluate inhalable delivery systems to manage respiratory infections [197]. Inhalable formulations for treating drug-resistant TB represent the least explored area of drug delivery for clinical validation. Only one dry powder inhalation formulation containing capreomycin has achieved a milestone, phase I, for treating drug-resistant tuberculosis. If demonstrated effectively, dry powder capreomycin inhalers could substantially enhance MDR-TB treatment by broadening its application in different clinical settings, including community-based, resource-limited, and supervised self-administration settings. The application of inhalable delivery technologies in pediatric drug-resistant TB is also appealing. However, additional research is required to formulate and evaluate dry powder versions of tuberculosis medications, which cannot be overstated [197,198].

LZD has demonstrated potential as a good option for inclusion in the treatment regimen for MDR-TB. However, it exhibits concentration-dependent pharmacokinetics, efficacy in killing *Mtb*, acquired resistance, and associated toxicity. Concentration-dependent efficacy and toxicity could be effectively regulated by administering a high dose of linezolid to the pulmonary tissues in which *Mtb* is located. Developing orally inhaled dry powder formulations as adjunctive therapy may offer an effective solution for TB patients and reduce treatment duration [197,199].

Rudolph and colleagues successfully developed an amorphous drug NP for inhalation therapy to target MDR-TB [200]. The NP contained BDQ and 1,3-benzothiazin-4-one 043 (BTZ) and was designed to overcome the lipophilicity of these drugs, which poses a challenge when used in aqueous environments. The lipophilic TB antibiotics were included in an elevated dose in the amorphous nanoparticles using a solvent–antisolvent technique for site-specific delivery in granulomas in *Mtb*-infected lungs. Importantly, minimal BDQ/BTZ nanoparticles were detected in the spleen or liver, indicating that drug targeting was focused. Animal studies using the pulmonary delivery route demonstrated the enhanced efficacy of the nano-formulations compared with those using nonformulated drugs. This successful technology yielded promising results, suggesting that the pulmonary delivery of BDQ/BTZ in NP could significantly improve the treatment of tuberculosis in the future [200].

BDQ-encapsulated fucosylated and nonfucosylated liposomes (BDQ-Lipofuc/BDQ-Lipo) were developed for intranasal delivery [201]. The pharmacodynamic and pharmacokinetic studies conducted in mice revealed that the intranasal delivery of liposomal BDQ formulations at a dose of 5 mg/kg BDQ and administered on alternate days for 14 days resulted in a substantial decrease in the lung burden of *Mtb* and an elevated concentration of BDQ in the lung when compared with BDQ delivered via the oral or intravenous routes of administration [201]. These findings suggest that a liposomal BDQ inhalation suspension could significantly improve anti-tubercular efficacy, suggesting that further exploration and development of this promising new treatment for tuberculosis is needed.

The poor aqueous solubility and absorption of DLM pose considerable challenges to achieving efficient oral administration [202]. To address this therapeutic hurdle, a self-microemulsifying DDS designed for administration via a pressurized metered dose inhaler (pMDI) to treat MDR-pulmonary tuberculosis was developed [202]. The performance of the self-microemulsifying drug delivery system (SMEDDS)-pMDI was evaluated using a next-generation impactor, and the ability to transport and deliver the medication to the innermost regions of the pulmonary system was demonstrated. Cellular internalization tests revealed effective delivery of the formulations into macrophage cells, suggesting the capability of achieving intracellular antimycobacterial activity. These results underscore the promise of using DLM-SMEDDS-pMDI to enhance the efficacy of DLM in treating MRD-TB and offer a bright outlook for the future of tuberculosis treatment [202].

All-trans-retinoic acid (ATRA) is an active and potent metabolite of vitamin A that has exhibited significant in vitro and in vivo efficacy against TB [128]. In response to the increasing global challenge of treating MDR-TB, Bahlool et al. [203] successfully developed a targeted treatment approach using ATRA-loaded nanoparticles (NPs) designed for nebulization, which is a potential new approach for the effective treatment of TB. Targeted inhaled HDT may provide a novel adjunctive treatment option for tuberculosis, with the potential to significantly improve existing dosage regimens, thereby improving the outcomes of MDR-TB cases, which may lead to improved patient outcomes and a reduction in the incidence of MDR-TB, offering a ray of hope in the fight against TB.

#### Dry Powder Inhalers (DPI)

DPI offers ease of use and is ideal for high-dose formulations to administer medications as a dry powder inhaled directly into the lungs for local and systemic activity. The propellant-free design enhances patient adherence and allows for high drug loading and improved stability, providing a reliable patient solution. The formulations can be engineered to incorporate either single drugs or combination therapies for the treatment of MDR-TB. They offer significant practical advantages in Sub-Saharan environments, primarily due to their enhanced portability, stability, and lack of reliance on refrigeration. Nonetheless, the efficacy of DPI is contingent on several factors, including the design of the inhaler, the powder formulation, and the airflow during inhalation. The performance of DPI has seen significant enhancement over the past ten years and is attributed to the incorporation of engineered nanoparticles and advancements in formulation technologies [19,194].

Inhalation formulation strategies encompass different approaches, including the use of liposomes, proliposomes, solid lipid nanoparticles, microemulsions, and metallic nanoparticles made of gold, silver, and zinc in addition to microparticles, polymeric nanoparticles, and dendrimers [194,197]. Liposomes have been the subject of extensive research and have been thoroughly investigated for pulmonary application. Numerous studies have highlighted their effectiveness in treating pulmonary conditions, providing a strong foundation to evaluate their potential for lung delivery with distinct benefits due to the inclusion of phospholipids, which are similar to naturally occurring pulmonary surfactants [204].

The potential of pulmonary administration of low drug doses, alongside reduced oral doses of the same substances, to significantly improve patient outcomes has been reported [205]. By formulating dry powder inhalation (DPI) preparations containing sutezolid (SUT), a second-generation pretomanid analog TBA-354 (TBA), or a fluorinated derivative of TBA-354 (32,625) within a matrix of the biodegradable polymer poly(L-lactide), a promising new approach was evaluated and revealed that oral doses of 100 mg/kg/day and DPI doses of 0.25–0.5 mg/kg/day of SUT, TBA-354, or 32,625 over 28 days were inadequately efficient in diminishing the lung and spleen burden of *Mtb* in infected mice. It was concluded that the incorporation of inhaled second-line medications as adjunct therapy could allow for a reduced oral dose for MDR-TB, offering hope for improved patient outcomes and adherence [205].

Despite the significant clinical effectiveness of LNZ against drug-resistant TB, safety and tolerability issues prompted Makled et al. [206] to formulate inhalable LNZ nano-embedded microparticles in a dry powder form. LNZ was integrated into non-structured lipid carriers (NLC), and the capability of LNZ-NLC to traverse mucosal barriers and infiltrate alveolar macrophages (AM, MH-S cells) was evaluated. The dried powder inhalation of LNZ-NLC in microparticles demonstrated sustained release of LNZ, enhanced mucus penetrability, and promises of safety at therapeutic doses, in vitro and in vivo targeting of macrophages, and better deposition in the alveolar regions of the lung. The favorable results, including the potential for less frequent administration, depend on lower doses and enhanced safety, which mitigates life-threatening systemic side effects [206].

PA-824 exhibits significant effectiveness against both active and dormant types of *Mtb* and is recognized for additive benefits when combined with PZA and MOX [207]. An inhalable combination powder formulation for the treatment of latent and MDR-TB was developed [207] with a high-dose fixed-dose combination (FDC) DPI formulation with elevated aerosolization efficiency, incorporating PZA and PA-824, or PZA and MOX, for targeted delivery to the alveolar regions of the lung where latent and resistant *Mtb* persists. PA-824 and MOX exhibited better stability in the combination powders than PZA. Additional research is required to enhance the stability of PZA in FDC powders intended for inhalation [207].

Administering inhalable formulations of repurposed drugs or HDT, with or without autophagy modulation, as an adjunct in the treatment of drug-resistant TB may alleviate the challenges associated with oral delivery by delivering drugs directly to the vicinity of *Mtb* within the interior granuloma of the infected lung regions. Pulmonary drug delivery systems used to treat active and MDR-*Mtb* strains are summarized in Table 4.

### 4.4. Combination Delivery Systems

The treatment of MDR-TB necessitates the administration of different drugs concomitantly to address the resistance effectively. In 2022, the WHO recommended a six-month course of therapy consisting of BDQ, PA-824, 600 mg of LZD, and MOX (BPaL) as the preferred treatment option over a nine-month or eighteen-month regimen for patients with MDR/rifampicin resistance-TB. Nanotechnology offers the capability to integrate these medicines into a single dose, thereby reducing the frequency of administration needed. Systems for drug delivery, which are engineered to deliver different anti-TB agents simultaneously, can enhance the effectiveness of combination therapy while minimizing the likelihood of resistance emerging. These systems may be nanoparticles such as liposomes or other drug nanocarriers [139,208].

The combined administration of antimicrobial agents that engage with different targets offers significant potential to reduce the likelihood of the development of resistance. This approach not only restores the sensitivity of multidrug-resistant bacteria to previously ineffective antibiotics but also reduces harmful side effects and allows for comparable antimicrobial effectiveness with reduced drug doses [209]. Integrating antibiotics with compounds that enhance or revive antibiotic efficacy against multidrug-resistant bacteria could revolutionize the treatment of MDR-TB using traditional anti-tubercular medications like RIF and INH.

#### Fixed-Dose Combination (FDC) Formulations

FDC formulations integrate multiple drugs into a single dosage form, streamlining the treatment process and enhancing patient adherence [210,211].

Typically, FDC has one or more of the following objectives, viz., (i) reducing the rate of acquired resistance through the use of drug combinations that exhibit minimal cross-resistance, thereby making the emergence of resistance dependent on the occurrence of multiple mutations in rapid succession which is unlikely, (ii) decreasing doses of drugs that have non-overlapping toxicity and comparable therapeutic profiles to improve efficacy while minimizing side effects, (iii) increasing cell sensitivity to drug effects through the administration of a different drug for chemosensitization or radiation for radiosensitization, by modifying the stages or growth characteristics of the cell cycle or cytokinetic optimization and (iv) achieving greater potency by leveraging additivity or synergistic effects, in the biochemical activity of two or three drugs [211]. The formulation of FDC of drugs can be integrated into nanoparticles or microparticles to achieve sustained, prolonged, and/or extended drug release [210].

The future of successful MDR therapy lies in using FDC medications, and further investigations should be undertaken to discover and develop such FDC [211]. However, the challenge of funding meticulously designed clinical trials and advancing the development of viable formulations, particularly for off-patent pharmaceuticals, remains a significant policy issue [211].

## 5. Challenges in the Implementation of Novel Delivery Strategies

Nano-therapeutics and nano-pharmaceuticals offer promising opportunities for earlier and more accurate diagnoses, better targeting of therapies, fewer adverse events, and improved therapeutic monitoring. These benefits may significantly improve the quality of life, promote healthier aging, and maximize healthcare cost-effectiveness. However, the domain of nano-medicine is still in its infancy, with most research confined to laboratories and limited success in clinical trials or medical applications having been demonstrated [212]. Despite the potential of these drug delivery systems, several significant challenges must be addressed.

### 5.1. Manufacture and Scale-Up

The intricate formulation and preparation of nano-therapeutics pose challenges for large-scale manufacturing, often leading to increased costs. There are very few, if any, lipid-based nanomedicines for anti-tubercular drug delivery due to scalability issues, high patient costs, and the need for specialized manufacturing equipment [213].

One of the primary obstacles in scaling up nanomedicine manufacture is maintaining the technology’s consistent physicochemical properties, including but not limited to size and drug loading across batches. While pilot-scale processes can attain reproducibility using well-characterized nanoparticles, industrial-scale production often faces challenges in controlling the polydispersity of nanomaterials. Differences in the physical and chemical properties between batches of nanomaterials can complicate pharmaceutical development [214].

Nanomedicines are complex three-dimensional products that require sophisticated chemistry, manufacturing, and process control. The lack of extensive experience in nanoscale, multi-component systems within traditional pharmaceutical manufacturing environments further exacerbates these challenges. In addition, the high cost of raw materials and multistep production adds to the complexity and contributes to elevated costs, further deterring attempts at large-scale production of nanocarriers [212].

### 5.2. Safety and Biocompatibility

To achieve clinical success, drug carriers must be non-toxic and biocompatible over extended periods. However, the toxicity of some nanomaterials poses a significant challenge. Experimental studies have demonstrated that silver, gold, silica, and titanium nanoparticles may adversely affect drug coupling and delivery [215].

Although preclinical studies provide insight into the pharmacokinetics of nanotechnology-based drug delivery systems (NDDS), they fail to fully capture interactions in the human body and other biological systems. Clinical trials remain essential to determining the safety and efficacy of drugs, as findings from animal models and cell lines often differ significantly from human responses [216,217]. For instance, only inhaled capreomycin has, to date, exhibited tolerability in Phase I clinical trials [198].

### 5.3. Regulatory Approval

Advancements in nanotechnology and nanomedicine can revolutionize the diagnostics and treatment of different diseases. However, the clinical translation process is complex, resource-intensive, and time-consuming. Success requires collaboration with many stakeholders, resource mobilization, and rigorous study designs to meet regulatory standards. Preclinical and clinical testing must establish safety and efficacy comprehensively before approval can be granted [218,219].

### 5.4. Patient Adherence

Patient acceptability of nano-systems is critical to their successful implementation and use [218]. However, maintaining adherence to lengthy and complex treatment regimens is challenging. Addressing this issue requires comprehensive solutions, including minimization of adverse effects and enhancing patient involvement in development activities. A multi-level approach that includes patients, healthcare professionals, and healthcare systems is necessary to optimize adherence [220].

### 5.5. Cost-Effectiveness

Nanotechnology-based solutions face significant budgetary constraints. Developing new pharmaceuticals for global health is costly, and reformulating existing drugs in nanocarriers may be a cost-effective alternative with a better chance of demonstrating efficacy and safety [218]. However, some nano-systems, such as liposomal amphotericin B, are prohibitively expensive for use in low-resource settings [221], and the cost of excipients, specific synthetic procedures, and storage conditions poses practical challenges, especially in the developing world [208,218].

### 5.6. Stability Issues

Nanoparticles and systems are prone to instability that manifests as agglomeration, disintegration, or premature drug release. Storage factors, including temperature, humidity, and exposure to light, may further compromise stability [214]. While novel drug delivery systems can transform the treatment of MDR-TB by improving drug bioavailability, targeting sites of infection, and reducing side effects, overcoming technical, safety, stability, regulatory, and logistical barriers is crucial. Rapid nanotechnological advancements, as seen during the SARS-CoV-2 pandemic, highlight the potential for translating laboratory innovations into practical healthcare solutions. Addressing these challenges through focused research and development may enable similar successes in advancing the treatment of patients with MDR-TB [218].

## 6. Future Directions

The battle against TB, particularly MDR-TB, requires innovative approaches to overcome existing challenges. Three critical areas for advancing immunomodulatory nanoparticle-based treatments have been identified and include addressing MDR infections and co-morbidities such as HIV/TB and HDT. Combining nanotechnology solutions with immunomodulatory agents or autophagy-inducing compounds (AICs) within an HDT framework may improve treatment outcomes. Such approaches may reduce drug resistance, dosing frequency, and costs while significantly enhancing the immune response of the host to TB infections, leading to better patient outcomes [121].

One promising strategy involves combining BDQ or other novel drug candidates with autophagy-inducing agents, such as vitamin D_3_, and functionalized nanoparticles targeting novel mycobacterial pathways. Administration of these treatment options via the pulmonary route could further enhance the management of drug-resistant TB. Furthermore, combining these advanced therapies with existing anti-TB drugs may mitigate the emergence of drug resistance.

Emerging technologies, including artificial intelligence (AI) and machine learning (ML), also offer considerable promise in developing approaches to TB management. AI algorithms can track drug resistance, enhance diagnostic precision, and predict adverse reactions, treatment duration, and outcomes. The analysis of large datasets, including genetic and treatment history, may facilitate the development of personalized treatment plans using AI, thereby optimizing therapeutic regimens [222,223]. In addition, AI-powered tools, mobile applications, and reminder systems would allow the prediction and improvement of patient adherence patterns. By identifying individuals at risk of non-adherence, healthcare providers can proactively intervene to enhance compliance [220,224].

Telemedicine and digital adherence technologies are increasingly integrated with diagnostic tools to improve timely diagnoses, follow-up, and real-time therapeutic monitoring, particularly in remote areas. These tools allow automated reminders and better communication between patients and healthcare providers, improving treatment outcomes [225,226]. However, successfully implementing AI and ML in TB management necessitates addressing several challenges, including data availability and quality, training healthcare workers, integrating AI with existing healthcare systems, and ensuring algorithm interpretability and reproducibility. Furthermore, ethical considerations, adequate funding, community awareness, and supportive policies must be prioritized to fully harness the potential of AI [222,225,226,227]. Incorporating AI technologies into routine clinical practice could revolutionize MDR-TB management. By enabling personalized treatment approaches, strengthening global TB control efforts, and facilitating adherence support, AI represents a transformative tool for improving patient outcomes and advancing efforts to eradicate TB.

## 7. Conclusions

Addressing multidrug-resistant tuberculosis (MDR-TB) demands a comprehensive approach that integrates innovative drugs, advanced drug delivery systems, and precision medicine development. Key research efforts should focus on developing and identifying functionalized nanocarriers to deliver fixed-dose combinations of existing anti-TB drugs, new therapeutic compounds, and host-directed therapies to enhance autophagy in the macrophages of the host. Although these strategies are vital, they are unlikely to be the sole solution to the challenges posed by MDR-TB. A future paradigm for TB treatment must incorporate a personalized approach that considers the genetics of different *Mycobacterium tuberculosis* strains and the host in combination with the drug susceptibility profiles for those strains. Furthermore, treatment regimens should address co-infections and comorbidities and tailor therapy for different forms of tuberculosis, including drug-susceptible TB, MDR-TB, latent TB, pre-extensively drug-resistant TB (Pre-XDR-TB), XDR-TB, and TDR-TB. Overcoming obstacles, such as balancing treatment efficacy with minimizing adverse effects and addressing cost barriers, is essential to improving global TB care and outcomes. As MDR-TB spreads rapidly, there is an urgent need for cost-effective, efficient treatment strategies and technologies. The ongoing advancements in MDR-TB research highlight the critical role played by scientists in developing accessible, personalized therapeutic strategies to combat this global health challenge.

## Figures and Tables

**Table 1:** Approved drugs for tuberculosis treatment, including mechanisms of action, targets, modes of administration, and key notes.

Drug Name	Mechanism of Action	Target	Mode of Administration	Key Notes	Refs
Isoniazid (INH)	Inhibits mycolic acid synthesis	Cell wall	Oral	Effective for active TB; resistance due to KatG mutations	[32,33]
Rifampin (RIF)	Inhibits RNA polymerase	RNA synthesis	Oral	Active against latent and active TB	[32,34]
Pyrazinamide (PZA)	Disrupts mycobacterial membrane metabolism	Fatty acid synthase pathway	Oral	Effective in acidic environments (e.g., phagolysosomes)	[27,35]
Ethambutol (EMB)	Inhibits arabinosyl transferase	Cell wall	Oral	Used to prevent resistance to other drugs	[31,36]
Streptomycin (STREP)	Inhibits protein synthesis (30S ribosome)	Ribosome	Intramuscular	Aminoglycoside requires injection; nephrotoxicity risk	[31,37]
Amikacin (AMI)	Inhibits protein synthesis (30S ribosome)	Ribosome	Intravenous	Used for MDR-TB; nephrotoxicity and ototoxicity risks	[32,38]
Capreomycin (CAP)	Inhibits protein synthesis	Ribosome	Intramuscular	Effective for MDR-TB; injectable; toxicity concerns	[31,39,40]
Kanamycin (KAN)	Inhibits protein synthesis (30S ribosome)	Ribosome	Intravenous	Aminoglycoside: alternative to amikacin	[30,31]
Moxifloxacin (MOX)	Inhibits DNA gyrase	DNA replication	Oral/Intravenous	More potent fluoroquinolone; risk of electrocardiographic QT interval prolongation	[31,40,41]
Gatifloxacin (GAT)	Inhibits DNA gyrase	DNA replication	Oral	Less commonly used; associated with glycemic changes	[31,42]
Levofloxacin (LEV)	Inhibits DNA gyrase	DNA replication	Oral	Fluoroquinolone is effective in resistance settings	[31,43]
Ofloxacin (OFL)	Inhibits DNA gyrase	DNA replication	Oral	Older fluoroquinolone; declining use	[31]
P-aminosalicylic acid (PAS)	Inhibits folate metabolism	Metabolism	Oral	Gastrointestinal side effects limit usage	[31]
Prothionamide (PTA)	Inhibits mycolic acid synthesis	Cell wall	Oral	Like Ethionamide, used for MDR-TB	[40]
Terizidone (TZD)	Inhibits cell wall synthesis	Cell wall	Oral	Alternative to cycloserine; less neurotoxic	[40]
Cycloserine (CYS)	Inhibits cell wall synthesis	Cell wall	Oral	Central nervous system toxicity limits the use	[32,40]
Ethionamide (ETH)	Inhibits mycolic acid synthesis	Cell wall	Oral	Used for MDR-TB; gastrointestinal side effects	[32,40]
Bedaquiline (BDQ)	Inhibits ATP synthase	Cell wall	Oral	Reserved for MDR/XDR-TB; QT prolongation risk	[27,30,40]
Delamanid (DLM)	Inhibits mycolic acid synthesis	Cell wall	Oral	Used for MDR/XDR-TB; well-tolerated; alternative to bedaquiline	[30,40]
Pretomanid (PA-824)	Generates reactive nitrogen species	Cell respiration	Oral	Effective in combination therapy	[40,44]
Linezolid (LZD)	Inhibits protein synthesis (50S ribosome)	Ribosome	Oral/Intravenous	Significant adverse effects; used in refractory TB	[30,40]
Clofazimine (CFZ)	Generates reactive oxygen species	DNA	Oral	Also used for leprosy, lipophilic compound	[32,40]
Rifapentine (RFT)	Inhibits RNA polymerase	RNA synthesis	Oral	Longer half-life; used in shorter-course treatments	[27]

QT-repolarization of the ventricles.

**Table 2:** Investigational drugs for tuberculosis treatment, highlighting mechanisms of action, targets, stages of development, and promising therapeutic potential.

Drug Name	Mechanism of Action	Target	Route	Stage	Key Notes	Refs
Telacebec (Q203)	Inhibits cytochrome bc1 complex	Respiration	Oral	Phase II	Promising for MDR-TB: reduces bacterial burden	[45]
Sutezolid (PNU-100480)	Inhibits protein synthesis (50S ribosome)	Ribosome	Oral	Phase II	Improved safety profile compared with Linezolid	[44,46]
Benzothiazinones	Inhibits DprE1 enzyme	Cell wall	Oral	Preclinical	Effective in drug-resistant TB strains	[47]
TBA-7371	Inhibits decaprenylphosphoryl-beta-D-ribose 2-epimerase	Cell wall	Oral	Phase I	Novel mechanism; active against MDR-TB	[48,49]
BTZ-043	Inhibits DprE1 enzyme	Cell wall	Oral	Phase II	Promising preclinical results	[50]
SQ109	Inhibits mycobacterial cell wall biosynthesis	Cell wall	Oral	Phase II	Synergistic with other TB drugs	[44,50]

**Table 3:** Summary of nanoparticle-based drug delivery system used in treating different strains of MDR-*Mtb*.

Drug Delivery System	Active Agent	Target	Refs
Liposomes	Zn(II) phthalocyanine (ZnPc)	(ATCC 27294) *Mtb* and MDR-TB (9037R)	[140]
Niosomes	Ethionamide	MDR-TB	[141]
Niosomes	Ethionamide	*Mtb* (H_37_RV)	[142]
Niosomes	Lipophilic ETH and hydrophilic D-Cycloserine	*Mycobacterium Smegmatis*	[143]
Polydopamine-coated silver nanoparticles	Rifampin	Multidrug-resistant strain of *Mtb*	[144]
Silver nanoparticles	Isoniazid	Resistant TB	[145]
Silver nanoparticles	Isoniazid	Clinical strain of resistant *Mtb*	[146]
Silver nanoparticles (AgNP) and zinc nanoparticles (ZnNP)	AgNP and ZnNP	*Mtb* and an MDR strain	[147]
Silver nanoparticles	AgNP	*Mycobacterium bovis* and *Mtb* H37Rv	[148]
Magnesium oxide nanoparticles (MnONP) and zinc oxide nanoparticles (ZnONP)	MnONP and ZnONP	H37Rv *Mtb* and MDR-*Mtb*	[149]
Silver nanoparticles and zinc oxide nanoparticles	AgNP and ZnONP	MDR and XDR-*Mtb*	[150]
PLGA nanoparticles	Moxifloxacin, econazole, and ethionamide	MDR-TB-infected mice	[151]
Alginate modified-PLGA nanoparticles	Amikacin and moxifloxacin	MDR-TB-*Mtb*-infected macrophages	[152]
PLGA-PEG Nanoparticle conjugated with the BM2 aptamer	Levofloxacin	*Mtb* model (Bacillus Calmette-Guérin bacteria (BCG))	[153]
Curdlan-functionalized PLGA Nanoparticles	Curdlan	Viable *Mtb* and MDR-TB	[154]
Mannose receptor-targeted bioadhesive chitosan Nanoparticles	Clofazimine	H37Rv *Mtb* strain	[155]
Multi-walled carbon nanotube nanofluid	Isoniazid and fluoxetine	Clinical strains of *Mtb*	[156].

**Table 4:** Pulmonary drug delivery systems used to treat active and MDR-*Mtb* strains.

Active Agent	Inhalation Formulation	Dosage form or Inhalation Device	Target	Refs
Bedaquiline and 1,3-benzothiazin-4-one 043 (BTZ)	Amorphous nanoparticles	Nebulization/nebulizer	Granulomas in *Mtb*-infected lungs	[200]
Bedaquiline	Fucosylated and nonfucosylated liposomes	Nebulization/nebulizer	*Mtb*-burden lung	[201]
Delamanid	Self-microemulsifying drug delivery system (SMEDDS)	Pressurized metered dose inhalation/inhaler	MDR-pulmonary tuberculosis	[202]
All trans-retinoic acid	PLGA nanoparticles	Nebulization/nebulizer	H37Ra avirulent *Mtb*	[203]
Sutezolid	Biodegradable polymer poly(L-lactide)-polymeric particles	Dry Powder inhalation/Inhalers	*Mtb*-burden lung and spleen	[205]
Linezolid	Non-structured lipid carriers (NLC) and microparticles	Dry Powder inhalation/Inhalers	*Mtb*-infected macrophages	[206]
Pretomanid, pyrazinamide and moxifloxacin	Spray-dried L-leucine powder	Dry Powder inhalation/Inhalers	Active, latent, and resistant *Mtb*-infected alveolar regions of the lung	[207]

## Data Availability

The original contributions featured in this study are incorporated in the article. Additional inquiries may be sent to the corresponding author.
